# Establishing the Temperature-Dependent Synthesis Window of β‑TaON by Coupling Predictive Thermodynamic Modeling with Experimental Validation

**DOI:** 10.1021/acs.chemmater.6c00139

**Published:** 2026-06-29

**Authors:** Aksha Gilbert Prince, Yuanchen Gao, Dmitri LaBelle, Jill K. Wenderott, Yong-Jie Hu

**Affiliations:** Department of Materials Science and Engineering, 6527 Drexel University, Philadelphia, Pennsylvania 19104, United States

## Abstract

Phase-pure synthesis has been a major challenge for transition-metal oxynitrides due to their sensitivity to synthesis conditions and limited understanding of their underlying thermodynamics. Beta-phase tantalum oxynitride (β-TaON), a promising candidate for (photo)­catalytic applications, is particularly difficult to reproducibly synthesize as a single-phase material at equilibrium. In this study, we developed and experimentally validated a thermodynamic model to identify optimal conditions for the single-phase synthesis of β-TaON via ammonolysis reactions. Gibbs free energies of reactant, product, and byproduct phases were predicted as a function of temperature using first-principles calculations with the quasi-harmonic approximation (QHA), as well as implemented from available thermodynamic databases. Utilizing these Gibbs energies, the calculation of phase diagrams (CALPHAD) approach was employed to develop a thermodynamic model for assessing the phase equilibria associated with the ammonolysis reactions, enabling prediction of temperature-dependent synthesis windows for β-TaON. Experimental syntheses were carried out across a range of temperatures and gas conditions, validating model predictions and iteratively refining the model accuracy through an integrated feedback loop. Co-flown gases beyond ammonia and water were also shown to be influential on β-TaON phase purity and reaction kinetics. A three-dimensional (3D) phase diagram predicted on the axes of parameters that can be controlled during practical synthesis quantitatively reveals a narrow, phase-pure synthesis window for β-TaON.

## Graphical abstract

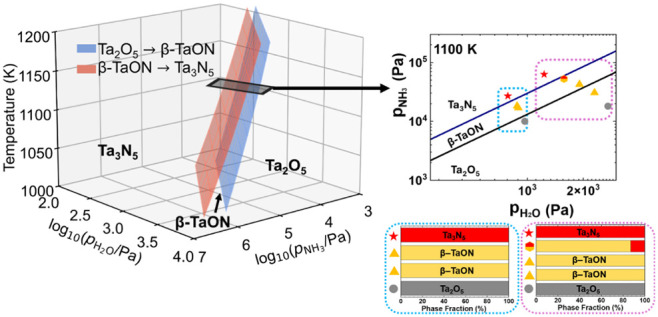

## 1 Introduction

Transition-metal oxynitrides, a subset of heteroanionic or mixed-anion materials, are a class of single-phase crystalline inorganic compounds containing both oxygen and nitrogen anions.^1,2^ Compared to oxygen, nitrogen is less electronegative and more polarizable, which increases the covalency of metal-anion bonding in oxynitrides. Hybridized *2p* orbitals in oxynitrides form shallower electronic bands relative to those in oxides, leading to a narrower and tunable band gap, often depending on the oxygen-to-nitrogen ratio.^3–5^ These combined effects give oxynitrides a unique set of electronic and optical properties, making them promising for applications in (photo)­(electro)­catalysis and (opto)­electronic and biomedical devices.^6–13^ As a notable example, the narrowed bandgap of oxynitrides, such as tantalum oxynitride (TaON), can enable visible-light-activated photocatalysis, as opposed to oxide photocatalysts that often require UV light to photogenerate charge carriers.^14–21^

Despite these advantages, synthesizing phase-pure oxynitrides remains challenging. The high bond energy of the nitrogen molecule (N_2_) and nitrogen’s low electron affinity result in higher formation energies for oxynitrides compared to oxides. As a consequence, even oxynitrides predicted to be thermodynamically stable often require precise control over nitrogen and oxygen activities to avoid decomposition, incomplete conversion, or formation of competing phases.^22^ A widely adopted synthesis method for oxynitrides is ammonolysis, during which oxide precursors react with flowing anhydrous ammonia (NH_3_) gas—often co-fed with hydrogen (H_2_) and water (H_2_O)—at elevated temperatures (∼500–1000 °C). Decomposition of NH_3_ is thermodynamically predicted to occur starting at ∼200 °C, but dissociation is kinetically slow even at higher temperatures (>500 °C) and dependent on flow rates (i.e., higher dissociation at lower flow rates).^22–25^ Thus, from a practical synthesis standpoint with high flow rates and high temperatures, undecomposed NH_3_ results in very high nitrogen activity, promoting incorporation of nitrogen into the anion sublattice.^25^ As alluded to previously, though, the exergonicity of ammonolysis reactions is highly sensitive to temperature and gas composition, making the synthesis of phase-pure oxynitrides nontrivial. Careful tuning of maximum reaction temperature, heating rate, gas flow ratios, and reaction time is required,^26^ and most reported syntheses rely on empirical experimental optimization.

TaON is a well-studied example of an oxynitride that is challenging to synthesize with high phase purity and reproducibility under equilibrium conditions. The orthorhombic phase of tantalum pentoxide (Ta_2_O_5_, space group P2mm^27^) is typically used as the precursor. The targeted ammonolysis reaction proceeds as follows:

1 $${\mathrm{Ta}}_{2}O_{5}+2{\mathrm{NH}}_{3}\left(g\right)=2\mathrm{TaON}+3H_{2}O\left(g\right)$$

Phase-pure β-TaON (monoclinic, space group *P*2_1_/*c* ^28^) synthesis requires tightly regulated nitrogen and oxygen activities. For example, low nitrogen activity or high oxygen activity can prevent ammonolysis from proceeding. Conversely, excessive nitrogen activity or insufficient oxygen activity can lead to over-nitridation to form Ta_3_N_5_ (orthorhombic, space group *Cmcm* ^29^) via:

2 $$3\mathrm{TaON}+2{\mathrm{NH}}_{3}\left(g\right)={\mathrm{Ta}}_{3}N_{5}+3H_{2}O\left(g\right)$$

eq 2 captures the scenario in which β-TaON, once formed from Ta_2_O_5_ as an intermediate, is further nitrided to Ta_3_N_5_ under excessively high nitrogen activity (see Figure S1). We do note that it is possible to form Ta_3_N_5_ directly from Ta_2_O_5_ under “dry” ammonolysis conditions (see Figure S1) as the synthetic window for β-TaON closes when the partial pressure of H_2_O (i.e., oxygen activity) goes to zero.

In practice, the nitrogen and oxygen activities are adjusted by co-feeding NH_3_ with controlled amounts of H_2_O (so-called “wet” ammonolysis) and often H_2_. However, due to limited understanding of reaction thermodynamics, previous optimization efforts have mostly relied on trial-and-error. As a result, synthesis conditions for β-TaON reported in the literature vary widely:^15,17,18,30–42^ maximum reaction temperatures ranging from 848 to 1273 K; gas flow conditions including NH_3_ (∼2–650 sccm), an oxygen source (e.g., H_2_O, O_2_), H_2_, and argon (Ar) bubbled through NH_4_OH; and reaction times of <1–68 h have been employed. It should be noted that in experiments with short reaction times β-TaON was not formed under thermodynamic equilibrium conditions, but rather as a reaction intermediate as Ta_2_O_5_ transformed into Ta_3_N_5_.^35,41^ Product-phase impurity challenges are also apparent, either from unreacted Ta_2_O_5_, over-nitridation to Ta_3_N_5_, or other metastable TaON polymorphs (γ and δ). The formation of these metastable polymorphs has been shown to be very sensitive to reaction conditions and precursor choice^43–46^ Clearly, the observed variations in synthesis conditions for β-TaON and the dearth of *thermodynamic equilibrium conditions* for synthesizing phase-pure β-TaON underscore the pressing need for a validated synthesis window that takes into account reaction temperature and oxygen and nitrogen activities.

To date, limited studies have explored the equilibrium synthesis window of β-TaON. Two reports that investigated the impact of NH_3_, H_2_, and H_2_O ratios on the formation of β-TaON were both conducted at a single reaction temperature: 1100 K.^30,31^ In 1972, Swisher and Read experimentally derived the phase boundaries between Ta_2_O_5_/β-TaON and Ta_3_N_5_/β-TaON via ammonolysis reactions of Ta_2_O_5_ powder samples, during which flow rates and ratios of NH_3_, H_2_, and H_2_O were systematically varied. Reaction times of 18–24 h were used to attempt to reach equilibrium products. In 2015, de Respinis et al.^31^ revisited this synthesis window by examining thin-film products of ammonolysis reactions, which were synthesized using a fixed H_2_O/H_2_ flow ratio while varying the flow of NH_3_. Their findings suggested that the viable synthesis window for β-TaON might be significantly narrower and may occur at lower nitrogen activities than proposed by Swisher and Read,^30^ though the influence of the products being thin films was cited as a possible reason for inconsistencies.^31^ The lack of consensus regarding the reported synthesis window for β-TaON, as well as the absence of data at different reaction temperatures, urges the need for a fundamental understanding of the underlying thermodynamics and phase equilibria governing the ammonolysis reactions, which can enable physics-based predictions of the synthesis window across multiple reaction temperatures.

In this work, we present an integrated computational–experimental framework that efficiently identifies the ammonolysis window for phase-pure β-TaON by systematically controlling the reaction temperature and reactive gas partial pressures. The thermodynamics and phase equilibria associated with the synthesis reaction are modeled by means of the calculation of phase diagram (CALPHAD) method.^47,48^ Additionally, first-principles calculations based on density functional theory (DFT) are coupled with the quasi-harmonic approximation (QHA) to provide necessary thermochemical input data for CALPHAD modeling. The model yields quantitative predictions of the equilibrium phase boundaries among the Ta_2_O_5_ precursor phase, the β-TaON target phase, and Ta_3_N_5_, a competing phase resulting from over-nitridation. Consequently, a three-dimensional (3D) synthesis window for β-TaON is outlined with respect to the reaction temperature and reactive gas partial pressures. A set of experimental ammonolysis reactions are conducted that precisely vary NH_3_, H_2_O delivered via Ar, and, optionally, H_2_ and N_2_ flow rates at two reaction temperatures in order to determine equilibrium product formation and validate model predictions. In addition to validation, the experimental results are subsequently fed back into the model as input data, iteratively enhancing its accuracy. The present work highlights the efficiency and effectiveness of responsive computational–experimental integration in identifying precise synthesis windows for inorganic compounds with complex chemistries and stringent synthesis conditions.

## 2 Materials and Methods

### 2.1 First-Principles Calculations

First-principles calculations based on DFT were performed using the projector augmented wave (PAW) method,^49,50^ as implemented in the Vienna Ab initio Simulation Package (VASP).^51^ The generalized gradient approximation (GGA) functional developed by Perdew–Burke–Ernzerhof (PBE)^52^ was employed to describe exchange–correlation interactions. The energy convergence criterion for electronic self-consistency was set as 10^–6^ eV for all calculations. A cutoff energy of 520 eV was used for the plane-wave basis for describing the electron wave functions. The first Brillouin zone sampling was performed using a 20 × 20 × 20 Γ-centered *k*-point mesh for β-TaON, while a 25 × 25 × 25 Γ-centered k-point mesh was used for Ta_3_N_5_. The input crystal structures of β-TaON and Ta_3_N_5_ were obtained from the Materials Project^53^ database, which have space group symmetries of *P*2_1_/*c* and *Cmcm*, respectively. These input structures were relaxed until the total energy difference between ionic steps fell below 10^–5^ eV. First-principles phonon calculations were performed using density functional perturbation theory (DFPT).^54^ 3 × 3 × 3 supercells of the relaxed unit cells were used for β-TaON and Ta_3_N_5_, while a 5 × 5 × 5 supercell of the primitive unit cell was used for bcc Ta. A *k*-point mesh of 5 × 5 × 5 was used for the phonon calculation of body-centered-cubic (bcc) Ta, while a 2 × 2 × 2 mesh was employed for the calculations of TaON and Ta_3_N_5_. In the relaxation calculations at fixed volumes for energy-volume (*E*–*V*) fitting, only atoms were allowed to relax, and the cell shape was kept fixed to preserve the crystal symmetry. The relaxation stops when the atomic forces are below 0.005 eV/atom. The Phonopy package^55^ was used for both pre- and post-calculation processing.

### 2.2 Prediction of Thermochemical Properties by the Quasi-Harmonic Approximation

Based on QHA, the Helmholtz free energy of a stoichiometric solid phase can be expressed as a function of volume (*V*) and temperature (*T*), given by,^48,56–58^

3 $$F\left(V,T\right)=E\left(V\right)+F_{\mathrm{vib}}\left(V,T\right)+F_{\mathrm{el}}\left(V,T\right)$$

where *E*(*V*) is the static energy at 0 K, *F*_vib_ is the free energy due to vibrational entropy, and *F*_el_ accounts for the entropic contribution from the thermal excitation of electrons. The static energy *E*(*V*) is a function of *V* and can be obtained by fitting DFT-calculated E-V data using a four-parameter Birch–Murnaghan equation of state (EOS),^59,60^

4 $$E\left(V\right)=a+bV^{-2/3}+cV^{-4/3}+dV^{-2}$$

where *a*,*b*,*c*, and *d* are fitting coefficients. From this fit, 0 K equilibrium properties, including the energy (*E*_0_), volume (*V*_0_), bulk modulus (*B*_0_), and its pressure derivative (*B*′) can be derived.

The vibrational free energy (*F*_vib_) is a function of *V* and *T*. At a given *V*, it can be derived from the phonon density of states (PDOS) (*g*(*ω*,*V*)),^61^

5 $$F_{\mathrm{vib}}\left(V,T\right)=k_{B}T\int_{0}^{\infty }\ln\left[2\sinh\frac{\hbar \omega }{2k_{B}T}\right]g\left(\omega ,V\right)d\omega$$

where *k*_B_ is Boltzmann’s constant, ℏ is the reduced Planck constant, and ω is the phonon’s frequency. In this work, the energy contribution due to thermal electron excitation (*F*_el_) is negligible because both TaON and Ta_3_N_5_ are semiconductors. The obtained Helmholtz free energy at the equilibrium volume (i.e., zero pressure) for each individual temperature was approximated as the Gibbs free energy for subsequent CALPHAD modeling because the *PV* contribution is negligible for solid phases at ambient pressure. Other thermodynamic properties, such as entropy (*S*), enthalpy (*H*), and heat capacity at constant pressure (*C*_P_), were derived from fundamental thermodynamic relations. In addition to TaON and Ta_3_N_5_, we also performed QHA calculations for the β-Ta_2_O_5_ phase but did not use the calculation results to model its Gibbs free energy function, since it was already available in the literature based on experimental measurements.^62,63^ Moreover, in Figure S2, we showed that the QHA-predicted Gibbs free energies for β-Ta_2_O_5_ are in good agreement with experiments.

### 2.3 CALPHAD Modeling

In the framework of the CALPHAD modeling, the Gibbs free energy of a stoichiometric solid phase is a function of temperature (*T*), which can be expressed as,^64–66^

6 $$G\left(T\right)-H^{\mathrm{SER}}=a+bT+cT\ln\left(T\right)+dT^{2}+eT^{-1}+fT^{3}$$

where *a, b, c*, *d*, *e*, and *f* are the coefficient parameters. *H* ^SER^ denotes the reference state of the Gibbs free energy and enthalpy. Specifically, CALPHAD uses the stable element reference (SER), in which the enthalpy of each pure element in its stable crystal structure at 298.15 K and 1 atm is defined as zero. Based on eq 6, *C*_p_, *S*, and *H* of the solid phase are given by,

7 $$C_{p}={\left(\frac{\partial H}{\partial T}\right)}_{P}=T{\left(\frac{\partial S}{\partial T}\right)}_{P}=-c-2dT-\frac{2e}{T^{2}}-6fT^{2}$$

8 $$S=-{\left(\frac{\partial G}{\partial T}\right)}_{P}=-b-c-c\ln\left(T\right)-2dT+\frac{e}{T^{2}}-3fT^{2}$$

9 $$H=G+TS=a-cT-dT^{2}+\frac{2e}{T}-2fT^{3}$$

For β-TaON and Ta_3_N_5_, the *C*_p_ values at and above room temperature obtained from the DFT-QHA calculations were fitted to determine the coefficients *c*,*d*,*e* and *f* using eq 7. The coefficient *b* was then determined from eq 8 using the entropy predicted by DFT-QHA at 298.15 K, since entropy is an absolute quantity.

However, the enthalpy predicted by DFT-QHA cannot be used directly to determine the coefficient *a*, because DFT and CALPHAD employ different enthalpy reference states. In DFT, the absolute energy depends on the choice of pseudopotentials and other computational settings. In CALPHAD, by the definition of SER, the enthalpy of a compound phase at 298.15 K is equivalent to its formation enthalpy with respect to the pure elements at the same temperature. Therefore, in this work, we used the formation enthalpy obtained by a reaction-based method $\left({\Delta H}_{f}^{\mathrm{rxn}}\right)$ to determine the coefficient *a* using eq 9. This approach was adopted because formation enthalpy derived from reactions within a chemically similar system was suggested to be generally more reliable.^67^ Specifically, to calculate the formation enthalpies of β-TaON and Ta_3_N_5_, we considered the following two reactions:

10 $${Ta}_{2}O_{5}+3\mathrm{TaN}+N_{2}\to 5TaON$$

11 $$N_{2}+3\mathrm{TaN}\to {Ta}_{3}N_{5}$$

Taking β-TaON as an example, to determine its formation enthalpy, 0 K DFT calculations were first performed to obtain the reaction enthalpy of eq 10. While DFT and CALPHAD employ different enthalpy reference states, the relative enthalpy difference between phases remains the same. Therefore, the reaction enthalpy derived from DFT energies must be equal to that derived from enthalpies expressed in the CALPHAD reference state, namely, ${\Delta H}_{\mathrm{rxn}}^{\mathrm{DFT}}\left(\mathrm{TaON}\right)={\Delta H}_{\mathrm{rxn}}^{\mathrm{CALPHAD}}\left(\mathrm{TaON}\right)$ . Because the experimentally measured formation enthalpies of TaN and Ta_2_O_5_ at 298.15 K are already available from the Scientific Group Thermodata Europe (SGTE) Substances version 6 (SSUB6) database and literature,^63,68^ respectively, the formation enthalpy of β-TaON per formula can then be derived as:

12 $$5{\Delta H}_{f}^{\mathrm{rxn}}\left(\mathrm{TaON}\right)={\Delta H}_{\mathrm{rxn}}^{\mathrm{DFT}}\left(\mathrm{TaON}\right)+\left({\Delta H}_{f}^{\mathrm{expt}}\left({\mathrm{Ta}}_{2}O_{5}\right)+3{\Delta H}_{f}^{\mathrm{expt}}\left(\mathrm{TaN}\right)\right)$$

Note that ${\Delta H}_{f}^{\mathrm{expt}}\left(N_{2}\right)$ does not appear in eq 12 because it is zero by its definition. Using the same approach, the formation enthalpy of Ta_3_N_5_ was derived based on eq 11. The derived ${\Delta H}_{f}^{\mathrm{rxn}}$ for β-TaON and Ta_3_N_5_ were taken as their enthalpy at 298.15 K to determine the coefficient *a* using eq 9.

Additionally, the formation enthalpies of β-TaON and Ta_3_N_5_ were also calculated using other conventional approaches, as discussed in detail in Tables S1 and S2 in the Supporting Information (SI). The results suggested that the formation enthalpy derived using the reaction-based method may serve as reasonable initial values for Gibbs free energy refinement based on the computation–experiment feedback loop described at the end of this section.

The Gibbs free energy of the reactive gas species as a function of temperature and partial pressure is adapted from the SSUB6 database.^68^ The SSUB6 database contains the Gibbs free energy functions for numerous gas species that were derived from high-quality experimental thermochemical data, such as the finite-temperature enthalpy and entropy of formation, and heat capacity. The SSUB6 database thus has been used to model phase stability and transformations involving gas molecules.^69,70^

Using the Gibbs free energy functions described above, Ellingham-type diagrams were constructed to evaluate the reaction equilibria among Ta_2_O_5_, β-TaON, and Ta_3_N_5_ as a function of temperature and the partial pressures of NH_3_ and H_2_O. Specifically, we considered two chemical reactions: (i) the targeted ammonolysis reaction described by eq 1, and (ii) an over-nitridation reaction leading to Ta_3_N_5_ described by eq 2. To determine the reaction equilibrium at a given temperature (*T*), the molar Gibbs free energies of the solid phases were calculated using eq 6. The chemical potentials of NH_3_ and H_2_O were derived as a function of their partial pressure (*P*_*i*_) assuming ideal gas behavior,

13 $$\mu_{i}\left(T,P_{i}\right)=\mu_{i}^{0}\left(T\right)+RT\ln\left(\frac{P_{i}}{P^{o}}\right)$$

where *P* ^o^ = 1 atm and $\mu_{i}^{o}\left(T\right)$ is the standard state chemical potential adopted from the SSUB6 database. The phase boundaries shown in the Ellingham-type diagram at a given *T* were obtained as a function of $P_{H_{2}O}$ and $P_{{\mathrm{NH}}_{3}}$ by finding their values that enforce a zero Gibbs energy of reaction for eq 1 and eq 2.

The partial pressures of individual gas species were further quantitatively linked to their volumetric flow rates, which were parameters directly controlled in our synthesis experiments. Specifically, before mixing in the reactive tube, for each flowing gas species *i*, where *i* is Ar, H_2_O, NH_3_, H_2_, or N_2_, we have,

14 $$PV_{i}=n_{i}RT$$

where *V_i_* is the volumetric flow rate, *n_i_* is the moles of *i* that flowed into the reactive tube during a unit time, and *P* is the pressure of *i* before mixing, which equals 1 atm for every gas species. After mixing in the reactive tube, the total pressure of the mixed gas *P*_total_ was still 1 atm, and for each species *i*, its partial pressure *P_i_* was derived as,

15 $$P_{i}=y_{i}P_{\mathrm{total}}$$

where *y_i_* is the mole fraction of *i* in the mixed gas. By its definition, *y_i_* was derived as,

16 $$y_{i}=\frac{n_{i}}{\underset{i}{\sum }n_{i}}$$

Combining eqs 14–16, we then have,

17 $$P_{i}=P_{\mathrm{tot}}\frac{V_{i}}{\underset{i}{\sum }V_{i}}$$

When NH_3_ is co-fed with H_2_O and H_2_ during ammonolysis, the equilibrium of the following two reactions can be reached among the gas species,

18 $$H_{2}O⇌\frac{1}{2}O_{2}+H_{2}$$

and

19 $${\mathrm{NH}}_{3}⇌\frac{1}{2}N_{2}+\frac{3}{2}H_{2}$$

At equilibrium, the activities of the NH_3_, N_2_, H_2_, H_2_O, and O_2_ species ( $a_{{\mathrm{NH}}_{3}},a_{N_{2}},a_{H_{2}},a_{H_{2}O}$ , and $a_{O_{2}}$ ) in the gas phase should satisfy,

20 $$K_{1}=\frac{a_{H_{2}}a_{O_{2}}^{1/2}}{a_{H_{2}O}}$$

and

21 $$K_{2}=\frac{a_{H_{2}}^{3/2}a_{N_{2}}^{1/2}}{a_{{\mathrm{NH}}_{3}}}$$

where *K*_1_ and *K*_2_ are reaction constants. Under the ideal gas assumption, the activity of a gas species is equivalent to its mole fraction. Based on eqs 20 and 21, we arrive at

22 $$a_{O_{2}}={\left(K_{1}\frac{y_{H_{2}O}}{y_{H_{2}}}\right)}^{2}\mathrm{and}\;a_{N_{2}}={\left(K_{2}\frac{y_{{\mathrm{NH}}_{3}}}{y_{H_{2}}^{3/2}}\right)}^{2}$$

Threfore, the phase boundaries in previous studies were plotted with respect to $\frac{y_{{\mathrm{NH}}_{3}}}{y_{H_{2}}^{3/2}}$ and $\frac{y_{H_{2}O}}{y_{H_{2}}}$ , as these two parameters provide quantitative measures of the effective oxygen and nitrogen activities, respectively. To compare the predicted phase boundaries with prior reports,^30,31^ $\frac{y_{{\mathrm{NH}}_{3}}}{y_{H_{2}}^{3/2}}$ vs $\frac{y_{H_{2}O}}{y_{H_{2}}}$ plots are also employed herein. The phase boundaries of the $\frac{y_{{\mathrm{NH}}_{3}}}{y_{H_{2}}^{3/2}}$ vs $\frac{y_{H_{2}O}}{y_{H_{2}}}$ plots were determined by solving the chemical potentials of NH_3_ and H_2_O that lead to a zero Gibbs free energy of reaction at a fixed temperature. Taking the phase boundary between Ta_2_O_5_ and β-TaON as an example, the reaction Gibbs free energy of eq 1 can be written as,

23 $${\Delta G}_{\mathrm{rxn}}\left(T,P\right)=G_{\mathrm{TaON}}\left(T\right)+\frac{3}{2}\mu_{H_{2}O}\left(T,P\right)-\frac{1}{2}G_{{\mathrm{Ta}}_{2}O_{5}}\left(T\right)-\mu_{{\mathrm{NH}}_{3}}\left(T,P\right)$$

At a fixed temperature, Gibbs free energy of solid phases is invariant, and eq 23 can thus be written as,

24 $$\begin{array}{ll} {\Delta G}_{\mathrm{rxn}} & =\left[G_{\mathrm{TaON}}\left(T\right)+\frac{3}{2}\mu_{H_{2}O}^{0}\left(T\right)-\frac{1}{2}G_{{\mathrm{Ta}}_{2}O_{5}}\left(T\right)-\mu_{{\mathrm{NH}}_{3}}^{0}\left(T\right)\right]\\ & +\mathrm{RT}\ln\left[\left(\frac{P_{H_{2}O}^{3/2}}{P_{{\mathrm{NH}}_{3}}}\right){\left(P^{0}\right)}^{-1/2}\right]\\ & =\mathrm{constant}+\mathrm{RT}\ln\left[\left(\frac{P_{H_{2}O}^{3/2}}{P_{{\mathrm{NH}}_{3}}}\right){\left(P^{0}\right)}^{-1/2}\right] \end{array}$$

Additionally, by assuming ideal gas behavior, we can rewrite $\frac{P_{H_{2}O}^{3/2}}{P_{{\mathrm{NH}}_{3}}}$ as,

25 $$\frac{P_{H_{2}O}^{3/2}}{P_{{\mathrm{NH}}_{3}}}{\left(P^{0}\right)}^{-1/2}=\frac{y_{H_{2}O}^{3/2}}{y_{{\mathrm{NH}}_{3}}}={[\frac{y_{H_{2}O}}{y_{H_{2}}}]}^{3/2}\cdot {[\frac{y_{{\mathrm{NH}}_{3}}}{y_{H_{2}}^{3/2}}]}^{-1}$$

When Ta_2_O_5_ and β-TaON reach equilibrium at a given temperature, Δ*G*_rxn_ = 0. Combining eqs 24 and 25, we have

26 $$1.5\mathrm{RT}\ln\left(\frac{y_{H_{2}O}}{y_{H_{2}}}\right)-\ln\left(\frac{y_{{\mathrm{NH}}_{3}}}{y_{H_{2}}^{3/2}}\right)=constant$$

Therefore, the phase boundary between Ta_2_O_5_ and β-TaON is then determined by solving $\frac{y_{H_{2}O}}{y_{H_{2}}}$ and $\frac{y_{{\mathrm{NH}}_{3}}}{y_{H_{2}}^{3/2}}$ that satisfy eq 26. In the same way, we calculated the phase boundary between β-TaON and Ta_3_N_5_ based on eq 2.

Given the potential uncertainties in the thermochemical properties of β-TaON and Ta_3_N_5_ derived from first-principles calculations, the enthalpy and entropy coefficients (i.e., *a* and *b* in eq 6) of their Gibbs free energy functions were optimized by minimizing deviations between the predicted and experimentally observed phase equilibria. The optimization was performed through a computational–experimental feedback loop, in which model predictions guided experiments to efficiently sample critical and previously unexplored regions of the phase diagram, while the resulting experimental data were fed back into the model to further refine the Gibbs free energy coefficients. After a few iterations, a finalized thermodynamic description was obtained for accurately predicting the synthesis window of β-TaON.

### 2.4 Experimental Synthesis Methods

Bulk samples were prepared using temperature-programmed ammonolysis of Ta_2_O_5_ (99.99% trace metal basis, Thermo Scientific Chemicals). For the syntheses, 100 mg of Ta_2_O_5_ powder, previously ground for approximately 15 min using a pestle and mortar, was placed in a quartz boat inside a tube furnace at room temperature and subjected to varied gas flow rates of anhydrous NH_3_, ultrapure H_2_, and ultrapure Ar, which served as a carrier gas for H_2_O vapor and was bubbled through a custom-made humidifier. All gases were supplied by Airgas. The flow rates for the various gases were controlled by mass flow controllers (Aera FCR7800, Proterial America) and a gas channel controller (ROD-4A, Proterial America). The H_2_O vapor flow rate was determined by flowing Ar at specific flow rates and measuring the mass loss of H_2_O from the humidifier over several hours. This procedure was repeated to determine an average H_2_O vapor flow rate. The heating profile for ammonolysis, all while under the flow of gases, was as follows: (1) heat to 150 °C and hold for 30 min, (2) ramp to maximum reaction temperature at 5 °C/min and hold for 20 h (or 40 h if indicated otherwise), and (3) allow the furnace to cool naturally to room temperature. Ammonia mixed with hydrogen gas poses a flammability hazard, so in our experiments, all gases were flowed through a tube furnace in a controlled manner without the presence of a strong oxidizer.

### 2.5 Material Characterization

The structures of ammonolysis products were characterized with powder laboratory X-ray Diffraction (PXRD) (Miniflex, Rigaku) using Cu Kα radiation. Typical scan rates, step sizes, and scan ranges for PXRD were 1.25 °/min, 0.01°, and 10° to 60°, respectively. Prior to PXRD, the powder products were ground and loaded onto a standard quartz powder sample holder. Rietveld refinement of the PXRD data was performed using GSAS-II.^71^

## 3 Results and Discussion

### 3.1 First-Principles Phonon and Thermochemical Properties

First-principles phonon dispersion curves and PDOS at the 0 K equilibrium volume are shown in Figure 1a-1c for bcc Ta, β-TaON, and Ta_3_N_5_, respectively. As a benchmark, the calculated phonon dispersion of bcc Ta agrees well with the experimental measurements.^72^ No negative-frequency phonon modes are observed for β-TaON and Ta_3_N_5_, indicating both phases are dynamically stable at 0 K. Additionally, it is interesting to note that negative phonon frequencies appear in the dispersion of β-TaON when the volume is expanded to 1.06 times the 0 K equilibrium volume (Figure S3), suggesting its thermal instability at elevated temperatures. This observation is consistent with the established understanding that β-TaON is a low-temperature polymorph of tantalum oxynitride.^46^

**Figure 1:**
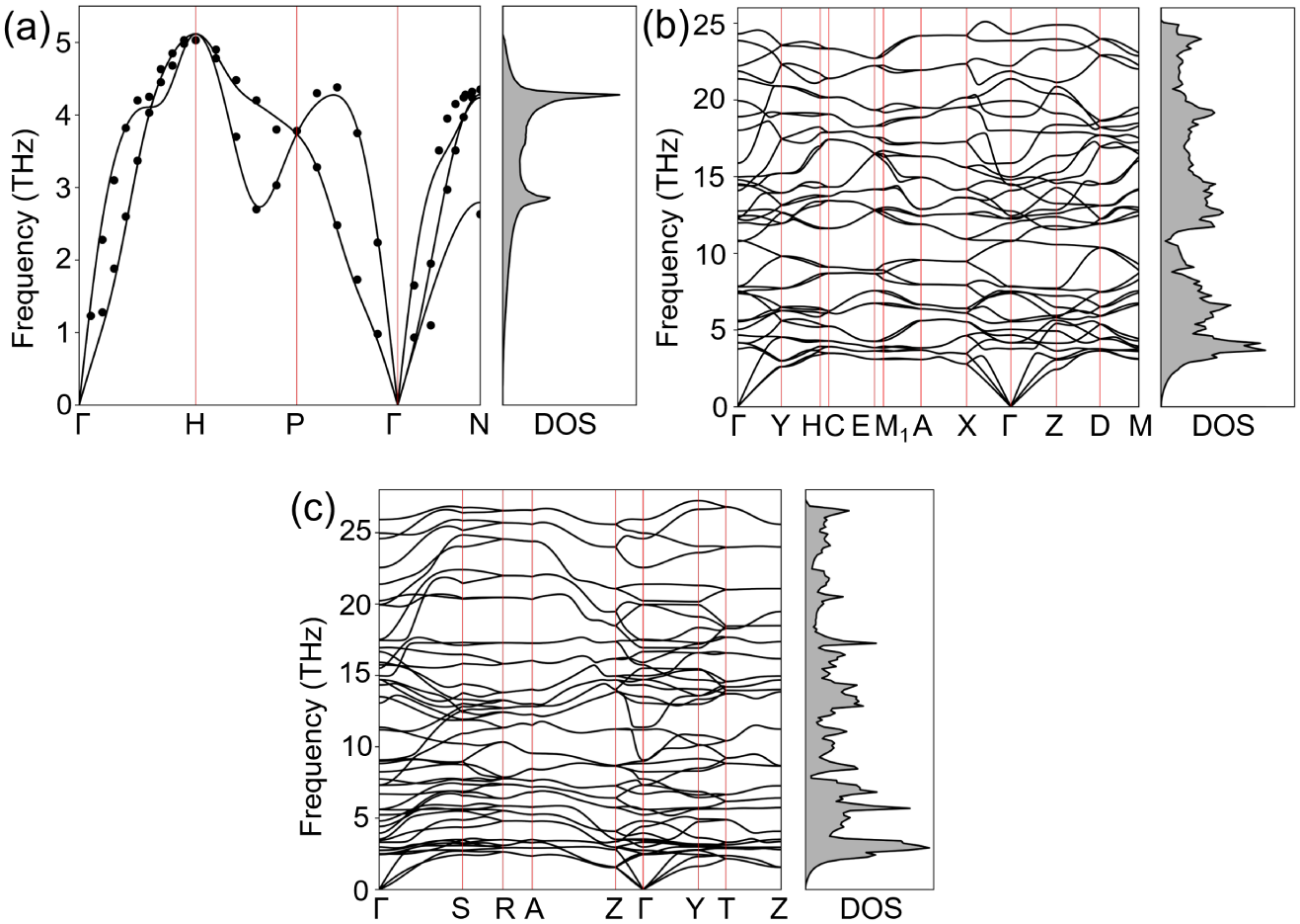
DFT-predicted phonon dispersion and density of states (DOS) for (a) bcc Ta, (b) β-TaON, and (c) Ta_3_N_5_ at their equilibrium volume. The solid dots represent the experimental measurements for bcc Ta.^72^

Figure 2 presents the DFT-QHA-predicted thermochemical properties, including heat capacity (*C*_P_), entropy (*S*), and Gibbs free energy (*G*), for pure Ta (Figure 2a-2c), β-TaON (Figure 2d-2f), and Ta_3_N_5_ (Figure 2g-2i) as a function of temperature. The predictions for pure Ta show good agreement with the experimental data from the Scientific Group Thermodata Europe (SGTE) database. This validates our computational approach and lends confidence to thermochemical properties predicted for β-TaON and Ta_3_N_5_, which lack experimental measurements. As described in Section 2.3, these temperature-dependent thermochemical data lead to the derivation of the Gibbs free energy functions of β-TaON and Ta_3_N_5_, based on which the Ellingham-type diagrams showcasing the synthesis window of β-TaON can be subsequently predicted.

**Figure 2:**
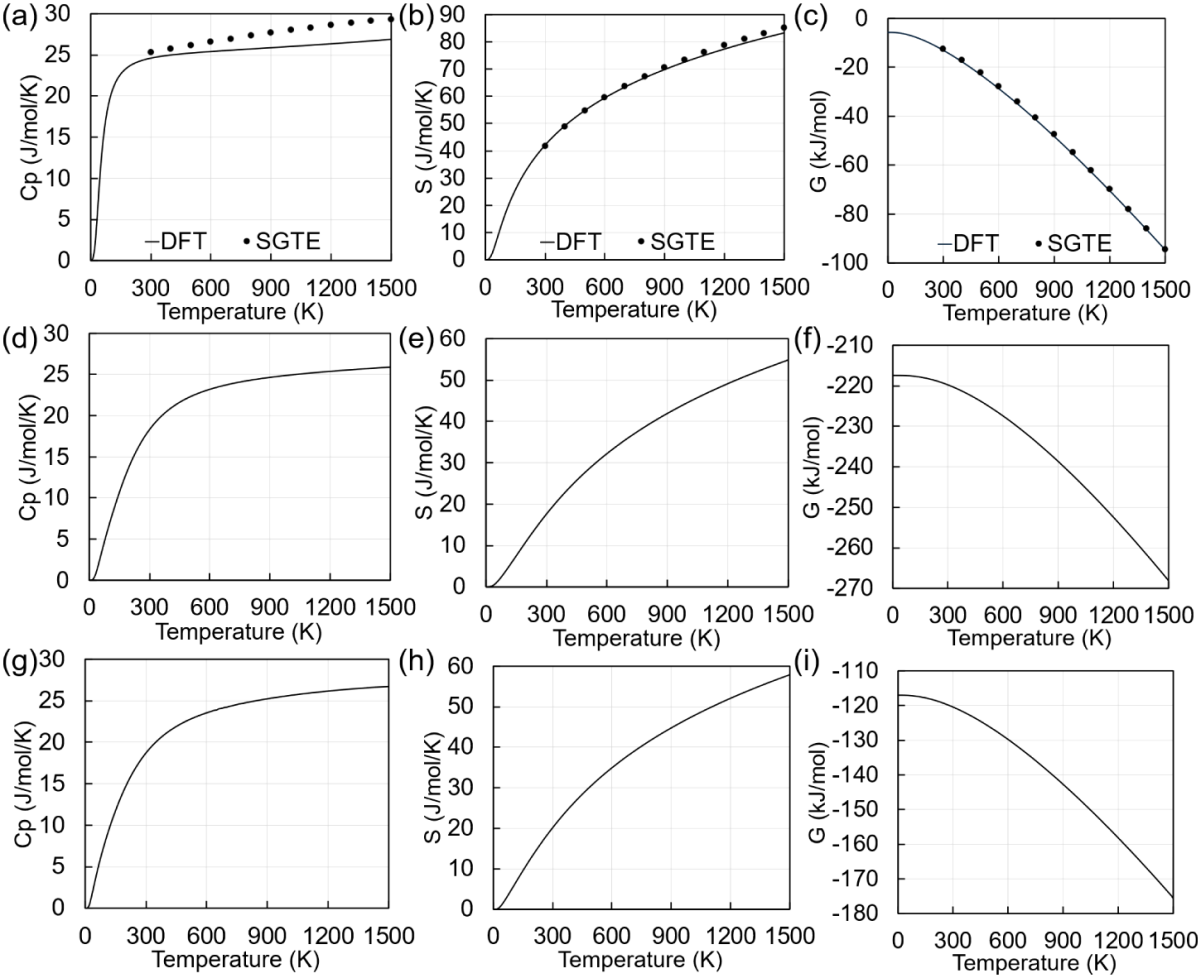
Temperature-dependent thermodynamic properties of bcc Ta, β-TaON, and Ta_3_N_5_ calculated via the quasi-harmonic approximation (QHA). (a–c) Thermodynamic properties for bcc Ta: Gibbs free energy *G*(*T*), entropy *S*(*T*), and heat capacity *C_p_* (*T*) compared against experimental values from the SGTE database, showing excellent agreement. (d–f) Thermodynamic properties for β-TaON, and (g–i) thermodynamic properties for Ta_3_N_5_.

### 3.2 DFT-Predicted Synthesis Window for β-TaON

Based on the Gibbs free energy functions of β-TaON and Ta_3_N_5_ purely derived from DFT calculations, we predicted phase boundaries between the single-phase regions of Ta_2_O_5_, β-TaON, and Ta_3_N_5_ at 1100 K to construct an initial estimate of the synthesis window of β-TaON. It should be noted that the Gibbs free energy function of Ta_2_O_5_ used for prediction was adopted from the literature,^62,63^ which was originally constructed based on high-fidelity experimental data. As shown in Figure 3, the phase boundaries were predicted as a function of $\frac{y_{H_{2}O}}{y_{H_{2}}}$ and $\frac{y_{{\mathrm{NH}}_{3}}}{y_{H_{2}}^{3/2}}$ for direct comparison with the experimental results reported by Swisher and Read^30^ and de Respinis et al.,^31^ and the hand-drawn phase boundaries by Swisher and Read.^30^ In Figure 3, the data points representing the synthesis results of Swisher and Read^30^ and de Respinis et al.^31^ were plotted from the raw gas flow rate data in the original articles, rather than taken directly from their published plots. We note that, in both studies, the ratio of gas flow rates, $\frac{V_{{\mathrm{NH}}_{3}}}{V_{H_{2}}^{3/2}}$ , was equated to the partial pressure ratio, $\frac{P_{{\mathrm{NH}}_{3}}}{P_{H_{2}}^{3/2}}$ , whereas, according to eqs 14–17, the correct relationship is actually $\frac{V_{{\mathrm{NH}}_{3}}}{V_{H_{2}}^{3/2}}=\frac{P_{{\mathrm{NH}}_{3}}}{P_{H_{2}}^{3/2}}{\left(\frac{P}{V}\right)}^{1/2}$ , where *P* and *V* are the total pressure and volumetric flow rate of the flowing gas mixture, respectively.

**Figure 3:**
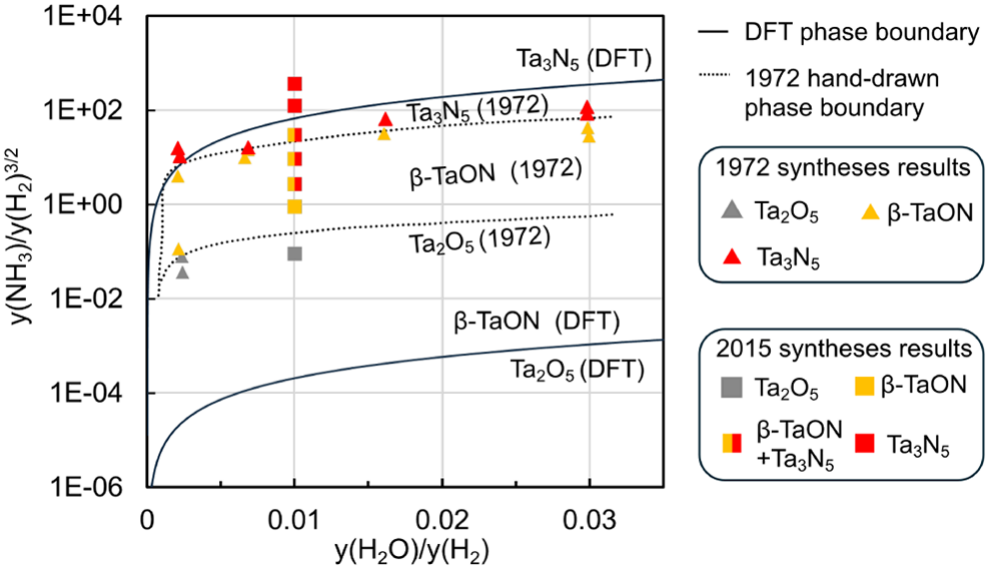
Synthesis window predicted using DFT-derived Gibbs free energy functions at 1100 K in comparison with literature results. The solid lines represent the DFT-predicted phase boundaries among Ta_2_O_5_, β-TaON, and Ta_3_N_5_, while the dashed lines represent the phase boundaries drawn by Swisher and Read in 1972. The synthesis results at varying gas conditions reported by Swisher and Read are marked by triangles, and those reported by de Respinis et al. are marked by squares. Gray, orange, and red symbols correspond to Ta_2_O_5_, β-TaON, and Ta_3_N_5_ phases, and the half-filled red symbol corresponds to a mixed β-TaON/Ta_3_N_5_ phase. Data reproduced from refs 30,31 .

As shown in Figure 3, using the DFT-predicted, unmodified Gibbs free energy functions of β-TaON and Ta_3_N_5_, our CALPHAD model predicts a wider synthesis window for β-TaON compared to the hand-drawn window reported by Swisher and Read in 1972.^30^ In particular, the lower window bound, which corresponds to the phase boundary between β-TaON and Ta_2_O_5_, is predicted to be around nitrogen activities much lower than those implied by the experimental results reported by Swisher and Read^30^ and de Respinis et al.^31^ This is unsurprising, as the DFT-predicted Gibbs free energies generally carry uncertainties on the order of 1 kJ/mol-atom, whereas phase boundary predictions within the CALPHAD framework can be sensitive to energy variations as small as 100 J/mol-atom. Therefore, the development of a reliable and robust CALPHAD-based thermodynamic model and database often requires elaborate efforts to further refine the DFT-predicted Gibbs free energy functions using experimentally measured phase equilibrium data.

Moreover, there is a clear inconsistency between the synthesis results of Swisher and Read^30^ and those of de Respinis et al.^31^ As shown by the semifilled squares in Figure 3, within the single-phase region of β-TaON proposed by Swisher and Read,^30^ de Respinis et al. obtained mixed products of β-TaON and Ta_3_N_5_. According to the Gibbs phase rule, a two-phase equilibrium in a three-component system at fixed temperature and total pressure has only one degree of freedom. Therefore, in a phase diagram with axes representing intensive thermodynamic variables, such as in Figure 3, the equilibrium between β-TaON and Ta_3_N_5_ can only appear as a line rather than a two-dimensional area. Since Ta_3_N_5_ is typically formed by over-nitridation of β-TaON, it is reasonable to conclude that, under the two-phase product conditions reported by de Respinis et al.,^31^ a single Ta_3_N_5_ phase is the actual equilibrium product and the two-phase mixtures are the result of reactions not achieving equilibrium. As such, the data of de Respinis et al.^31^ suggest a much narrower single-phase region (i.e., synthesis window) for β-TaON than that proposed by Swisher and Read.^30^ Furthermore, it is challenging to assess phase purity for β-TaON samples of Swisher and Read^30^ reported within the single-phase region due to the lack of published diffraction patterns: descriptions of the color of TaON samples (e.g., green, amber, and reddish-brown) varying across the phase field point to the possibility of significantly mixed-phase products. These discrepancies among reported experimental results as well as our DFT predictions urge the need for a new set of comprehensive, consistent synthesis trials, based on which our CALPHAD model, in particular the DFT-predicted Gibbs energy functions, can be refined and validated for robust prediction of the β-TaON synthesis window across varying temperature and gas flow conditions.

### 3.3 Accurate Construction of Synthesis Window via a Computational–Experimental Feedback Loop

To further refine and validate our CALPHAD model for accurate prediction of the β-TaON synthesis window, ammonolysis of the Ta_2_O_5_ precursor was performed at different temperatures with varying flow rates of NH_3_, H_2_O delivered via Ar, and, optionally, H_2_ and N_2_. Importantly, the model was iteratively improved via experiments in a feedback loop. At each iteration, the computational model first predicted the Ellingham-type diagram for β-TaON, Ta_2_O_5_, and Ta_3_N_5_ at a given temperature. The prediction then guided our experimental syntheses to efficiently sample the synthesis conditions critical for validating the predicted single-phase region of β-TaON and its phase boundaries with Ta_2_O_5_ and Ta_3_N_5_. The experimental results were subsequently used to modify the DFT-predicted Gibbs free energy functions of β-TaON and Ta_3_N_5_, updating the CALPHAD model to best reproduce the experimentally observed phase equilibria. The refined model was employed to predict the phase diagram at another temperature, which in turn directed the next round of experiments. This iterative process was repeated until the CALPHAD model produced finalized phase equilibrium predictions in agreement with experimental observations across different temperatures and reactive gas partial pressures (e.g., $p_{{\mathrm{NH}}_{3}}$ , $p_{H_{2}O}$ ). The predicted phase diagram was also validated with ammonolysis products synthesized using reaction conditions that replaced H_2_ with N_2_ as a coflown gas and without either H_2_ or N_2_ as coflown gases.

#### 3.3.1 Loop Iteration at 1050 K with Coflown H_2_ Gas

As hinted at in the literature, the β-TaON synthesis window appears to be quite narrow at 1100 K. From the model refinement perspective, a narrow synthesis window corresponds to fewer synthesis conditions to yield the single-phase product of interest and, thus, fewer data points upon which to refine predictions. Preliminary CALPHAD modeling pointed toward a wider window for practical synthesis of β-TaON at lower reaction temperatures, and so ammonolysis syntheses were conducted at 1050 K to first iteratively refine the model. Following the procedures of Swisher and Read and de Respinis et al.,^30,31^ NH_3_, H_2_O delivered via Ar, and H_2_ were coflown to vary the partial pressures of NH_3_ and H_2_O, thus affecting the thermodynamic spontaneity of the ammonolysis reactions described by eqs 1 and 2. The selection of gas flow rates was based on initial CALPHAD phase equilibria predictions at 1050 K. The selected gas flow rates and their resulting reactive gas partial pressures for each synthesis trial are summarized in Table 1. After ammonolysis, we found that products had ∼0% mass loss at conditions with relatively low $p_{{\mathrm{NH}}_{3}}$ , demonstrating that nitrogen activities at those conditions were not sufficient for nitriding Ta_2_O_5_. As the NH_3_ flow rate increased while fixing the H_2_ flow rate, mass loss of ∼3–5% was observed, indicating nitridation of Ta_2_O_5_ to TaON (predicted ∼4.7% mass loss). Mass loss of ∼8% was observed by further increasing the NH_3_ flow rate while fixing the H_2_ flow rate, and this corresponded to transformation of Ta_2_O_5_ to Ta_3_N_5_ (predicted ∼8.1% mass loss). The mass loss changes across different gas partial pressures support the expected transformation of oxide precursor to oxynitride and nitride, dependent based on the ammonolysis conditions.

**Table 1:** Lists the Gas Flow Ratios of Relevant Species and Observed Products and Phase Fractions of the Products Extracted from Diffraction Pattern Refinement (See SI for Diffraction Patterns and Refinement). Varying oxygen and nitrogen activities, as well as the maximum ammonolysis temperature, significantly impact the product phase(s) observed.

T (K)	Ar + H_2_O (sccm)	NH_3_ (sccm)	H_2_ (sccm)	N_2_ (sccm)	$p_{H_{2}O}$ (Pa)	$p_{NH_{3}}$ (Pa)	Product Phases
1050	124.5	100	11	0	1909	42,418	Ta_2_O_5_
	124.5	140	11	0	1632	50,771	36% Ta_2_O_5_ + 64% β-TaON
	124.5	180	11	0	1425	57,007	15% Ta_2_O_5_ + 85% β-TaON
	124.5	220	11	0	1265	61,841	95% β-TaON + 5% Ta_3_N_5_
	124.5	260	11	0	1082	67,348	23% β-TaON + 77% Ta_3_N_5_
	124.5	350	11	0	926	72,054	Ta_3_N_5_
	102.9	60	167	0	879	18,187	Ta_2_O_5_
	102.9	95	167	0	795	26,035	33% Ta_2_O_5_ + 67% β-TaON
	102.9	160	167	0	675	37,218	93% β-TaON + 7% Ta_3_N_5_
	102.9	177	167	0	649	39,606	3.3% γ-TaON + 94% β-TaON + 2.7% Ta_3_N_5_
	102.9	242	167	0	567	47,275	57% β-TaON + 43% Ta_3_N_5_
	102.9	350	167	0	468	56,461	Ta_3_N_5_
1100	124.5	30	11	0	2715	18,100	Ta_2_O_5_
	124.5	60	11	0	2299	30,651	95.5% β-TaON + 4.5% Ta_2_O_5_
	124.5	100	11	0	1909	42,418	96% β-TaON + 4% Ta_3_N_5_
	124.5	150	11	0	1575	52,493	47% β-TaON + 53% Ta_3_N_5_
	124.5	230	11	0	1230	62,885	Ta_3_N_5_
	102.9	30	167	0	967	10,003	Ta_2_O_5_
	102.9	55	167	0	893	16,928	75.3% γ-TaON + 24.7% β-TaON
	102.9	60	167	0	879	18,187	8.5% γ-TaON + 91.5% β-TaON
	102.9	100	167	0	784	27,034	Ta_3_N_5_
1050	124.5	100	0	11	1909	42,418	Ta_2_O_5_
	124.5	140	0	11	1632	50,771	12% Ta_2_O_5_ + 88% β-TaON
	124.5	180	0	11	1425	57,008	99.5% β-TaON + 0.5% Ta_3_N_5_
	124.5	220	0	11	1265	61,841	99.2% β-TaON + 0.8% Ta_3_N_5_
	124.5	260	0	11	1082	67,348	99.4% β-TaON + 0.6% Ta_3_N_5_
	124.5	350	0	11	926	72,054	Ta_3_N_5_
	102.9	60	0	167	879	18,187	Ta_2_O_5_
	102.9	95	0	167	795	26,035	β-TaON
	102.9	160	0	167	675	37,218	99.7% β-TaON + 0.3% Ta_3_N_5_
	102.9	177	0	167	649	39,606	99.5% β-TaON + 0.5% Ta_3_N_5_
	102.9	242	0	167	567	47,275	Ta_3_N_5_
	102.9	350	0	167	468	56,461	Ta_3_N_5_
1100	124.5	30	0	11	2715	18,100	Ta_2_O_5_
	124.5	60	0	11	2299	30,651	β-TaON
	124.5	100	0	11	1909	42,418	99.4% β-TaON + 0.6% Ta_3_N_5_
	124.5	150	0	11	1575	52,493	87% β-TaON + 13% Ta_3_N_5_
	124.5	230	0	11	1230	62,885	Ta_3_N_5_
	102.9	30	0	167	967	10,003	Ta_2_O_5_
	102.9	55	0	167	893	16,928	β-TaON
	102.9	60	0	167	879	18,187	99.7% β-TaON + 0.3% Ta_3_N_5_
	102.9	100	0	167	784	27,034	Ta_3_N_5_
1050	102.9	100	0	0	1429	49,285	27% Ta_2_O_5_ + 73% β-TaON
	102.9	150	0	0	1147	59,312	49% Ta_2_O_5_ + 51% β-TaON
	102.9	150	0	0	1147	59,312	^a^15% Ta_2_O_5_ + 85% β-TaON
	102.9	200	0	0	957	66,028	59% β-TaON + 41% Ta_3_N_5_
1100	102.9	55	0	0	1837	34,832	70% Ta_2_O_5_+ 30% β-TaON
	102.9	70	0	0	1677	40,486	13.5% Ta_2_O_5_ + 86.5% β-TaON
	102.9	70	0	0	1677	40,486	^a^0.9% Ta_2_O_5_+ 99.1% β-TaON
	102.9	100	0	0	1429	49,285	97.9% β-TaON + 2.1% Ta_3_N_5_

a Indicates reactions that were performed for 40 h. Otherwise, reactions were performed for 20 h under standard conditions discussed in the experimental methods.

Transformation of Ta_2_O_5_ precursor to TaON and Ta_3_N_5_ was further quantified with PXRD (see Figure 4a-4c for 1050 K trials). Ammonolysis product phases and phase fractions, extracted from Rietveld refinement of diffraction data (phase fractions shown in Figure 4b and 4c and refinement shown in Figures S4 and S5), are listed in Table 1 and plotted in Figure 4a based on their synthesis conditions. The observed phase transitions with varying $p_{{\mathrm{NH}}_{3}}$ and $p_{H_{2}O}$ are consistent with the mass loss analysis, namely that as $p_{{\mathrm{NH}}_{3}}$ increases and $p_{H_{2}O}$ decreases, the observed products are Ta_2_O_5_, followed by TaON, and then Ta_3_N_5_. This observation is consistent across reaction temperatures (1050 and 1100 K). More importantly, as shown in Figure 4, our synthesis results indicate that the phase stability of β-TaON is highly sensitive to changes in reactive gas partial pressures. The single-phase region for β-TaON appears confined to a narrow range of $p_{{\mathrm{NH}}_{3}}$ across $p_{H_{2}O}$ . It is noteworthy that secondary phases (often <5%) were observed under conditions that predominantly favor the formation of the β-TaON phase. To determine if kinetics were responsible for incomplete reactions, points within the β-TaON synthetic window were retested, and the reaction time was doubled from 20 to 40 h; at these extended reaction times, secondary phases (often Ta_3_N_5_) were still present at fractions typically less than 5% (see Figure S6a-S6c). Thus, the appearance of such secondary phases does not seem to be a kinetic effect. Furthermore, owing to the somewhat reproducible nature of the secondary phases, batch-to-batch variation is also not likely to blame.

**Figure 4:**
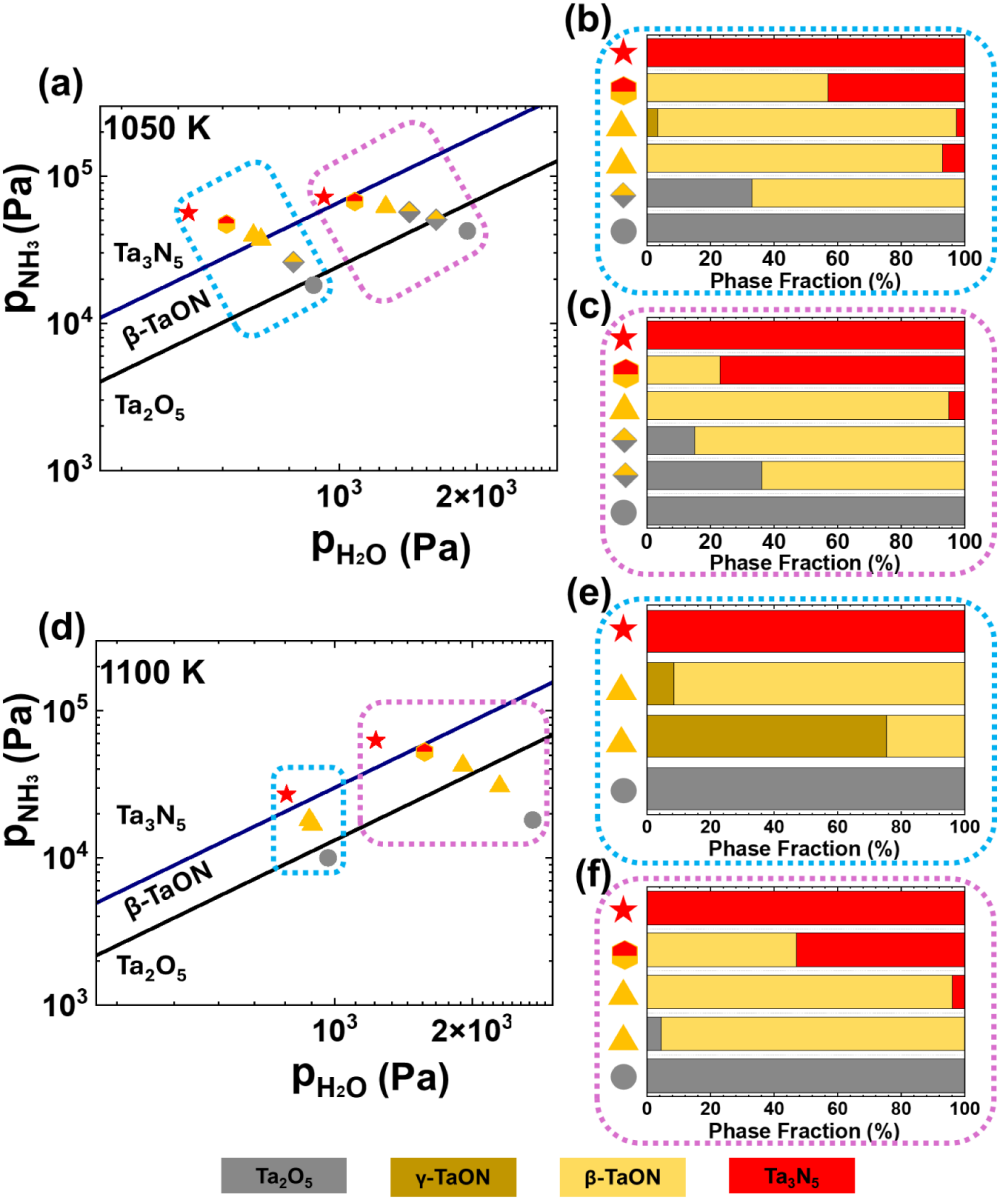
(a) Computationally predicted Ellingham-type diagram of β-TaON synthesis window at 1050 K with experimental synthetic validation (combined gas flow of Ar/H_2_O, H_2_ and NH_3_). (b), (c) Phase fractions of products (1050 K reaction temperature) at lower and higher water flow rates, respectively. (d) Computationally predicted Ellingham-type diagram of β-TaON synthesis window at 1100 K with a combined gas flow of Ar/H_2_O, H_2_ and NH_3_ with experimental synthetic validation. (e), (f) Phase fractions of products (1100 K reaction temperature) at lower and higher water flow rates, respectively. In (a) and (d), black and blue lines correspond to the Ta_2_O_5_/β–TaON and Ta_3_N_5_/β-TaON phase boundaries, respectively. Gray circles, yellow triangles, and red five-point stars denote majority (>90%) Ta_2_O_5_, TaON, Ta_3_N_5_ product phases, respectively. Dual-colored markers indicate the conditions yielding products containing two phases with significant amounts, where the gray/yellow dual-colored four-point diamond shapes denote Ta_2_O_5_/TaON two-phase products, while the red/yellow dual-colored hexagons denote a TaON/Ta_3_N_5_ mixed product. In (b), (c), (e), and (f), red, dark yellow, yellow, and gray colors correspond to Ta_3_N_5_, γ-TaON, β-TaON, and Ta_2_O_5_, respectively, and the symbols from bottom to top in each plot are in order of increasing partial pressure of NH_3_ while keeping water flow rate constant.

With the results of the ammonolysis syntheses, namely the phases and phase fractions present in the products, the CALPHAD model was further refined. Specifically, synthesis conditions that formed two phases with comparable fractions (>10% secondary phase) were treated as indications of two-phase equilibrium and used to pin the model-predicted phase boundaries, while conditions yielding a single predominant phase (>90%) were used to inform potential locations of single-phase regions. For this iteration, we adjusted only the *a* parameter in the Gibbs free energy functions (eq 6) of β-TaON and Ta_3_N_5_, corresponding to modifications of their enthalpies. The Gibbs energy function of Ta_2_O_5_ was derived from high-fidelity experimental data and was therefore left unchanged. We found that moderately increasing the *a* parameter by a few percent relative to its DFT values was sufficient to reconcile the model with experiment. As shown in Figure 4a-4c, the refined CALPHAD model reproduced not only the narrow single-phase region for β-TaON but also the β-TaON/Ta_2_O_5_ and β-TaON/Ta_3_N_5_ phase boundaries, largely agreeing with the phase equilibria and transitions indicated by the experimental syntheses.

#### 3.3.2 Loop Iteration at 1100 K with Coflown H_2_ Gas

The CALPHAD model refined with the 1050 K synthesis data was next applied to predict the phase equilibria at 1100 K, which was anticipated to show a narrower phase-pure synthesis window for β-TaON. This prediction guided the selection of ammonolysis conditions for another round of experimental trials. The selected gas flow rates and their resulting $p_{{\mathrm{NH}}_{3}}$ and $p_{H_{2}O}$ are summarized in Table 1, together with the product phase fractions. With the experimental results at 1100 K, a subsequent round of model refinement was carried out. Correspondingly, the entropy-related parameter (*b* in eq 6) of the Gibbs free energy functions for β-TaON and Ta_3_N_5_ was adjusted to reproduce the experimental results at both 1050 and 1100 K. Because entropy is temperature-dependent, incorporating synthesis results at multiple temperatures allowed for more reliable tuning of the *b* parameter. The *a* coefficients refined in the previous iteration at 1050 K also changed slightly in response to the *b* parameter adjustments. In the finalized model, which was used to predict the phase boundaries in Figures 4–6, the *a* parameter was increased by +20.8 kJ/mol-atom for β-TaON and +13.3 kJ/mol-atom for Ta_3_N_5_, which shifted their enthalpies by ∼10% and ∼8% relative to the DFT values, respectively. The adjustments of the *b* parameter were relatively minor, only −0.50 J/K/mol-atom for β-TaON and −0.35 J/K/mol-atom for Ta_3_N_5_, resulting in changes of less than 1% relative to the DFT-predicted values.

As shown in Figure 4d, the experimental and model-predicted phase equilibria at 1100 K are also in good agreement: predominant oxynitride products formed under conditions within the predicted single-phase region of β-TaON, and two-phase products were observed near the predicted phase boundaries (phase fractions shown in Figure 4e and 4f, garnered from diffraction pattern refinement shown in Figures S7 and S8). Notably, both our experimental and modeling results indicate that the single-phase region of β-TaON at 1100 K (the results plotted on the $\frac{y_{H_{2}O}}{y_{H_{2}}}$ and $\frac{y_{{\mathrm{NH}}_{3}}}{y_{H_{2}}^{3/2}}$ axes are shown in Figure S9) is considerably narrower than that proposed by Swisher and Read (dashed lines in Figure 3). As with the 1050 K experiments, we observed secondary phases (often <5%) at conditions that predominantly favor the formation of the β-TaON phase; reactions with doubled hold times from 20 to 40 h for points within the β-TaON synthetic window were again sampled, and such secondary phases were still present at fractions typically less than 5% (see Figure S6d and S6e).

Additionally, in Figure 4d, we note a discrepancy between experimental and computational results, where a majority oxynitride product was obtained below the predicted oxide/oxynitride phase boundary. This is possibly due to the presence of hydrogen in the oxynitride product, as observed in other ammonolysis-derived oxynitrides.^73^ In an Ellingham-type diagram such as Figure 4d, the slope of a phase boundary is intrinsically determined by the ratio of the stoichiometric coefficients of the gas species in the chemical reaction. For example, the slopes of the oxide/oxynitride and oxynitride/nitride phase boundaries in Figure 4d are the same, both equal to $\frac{3}{2}$ , because the stoichiometric coefficients NH_3_ and H_2_O are identical in eqs 1 and 2. Modifying the Gibbs free energy of the solid phases would only change the intercept of the phase boundaries rather than the slope. However, a smaller stoichiometric coefficient of H_2_O can be achieved in eq 1 if the formed oxynitride contains hydrogen, since some of the hydrogen in NH_3_ now goes into the oxynitride product. Consequently, the oxide/oxynitride phase boundary could possess a slope smaller than $\frac{3}{2}$ to possibly satisfy both sets of experimental results (i.e., at low and high $p_{H_{2}O}$ values), as seen in Figure 4d.

Another interesting phenomenon we observed in our 1100 K syntheses is the presence of metastable γ–TaON in competition with the β–TaON phase (see Figure 4d and 4e, blue rectangle). γ-TaON is a high-temperature polymorph of β–TaON with an enthalpy only ∼0.07 eV/atom higher than that of β–TaON at 0 K.^74^ Therefore, a higher synthesis temperature can considerably increase the driving force for the formation of γ-TaON to be competitive with β–TaON due to entropy contributions. There are reports in the literature of β–TaON and γ–TaON as mixed-phase products via ammonolysis at maximum reaction temperatures of 1123 K, but these syntheses are challenging to directly compare with our results as they involve significantly shorter reaction times (5–10 h).^46,75^ Furthermore, phase-pure γ–TaON has been achieved with ammonolysis, but again the reaction conditions are not easily comparable with short reaction time (3 h) and the use of an amorphous tantalum oxide precursor.^45,76^ We note that the polymorphic transformation temperature between these two phases has not been firmly established via experiments. Therefore, we were not able to include the γ-TaON phase in the current CALPHAD model for phase equilibrium prediction, given that DFT is generally not sufficiently accurate to reliably predict the transition temperature. Nevertheless, a recent work suggested a β-to-γ transition temperature between 1073 and 1123 K,^45^ close to our synthesis temperature of 1100 K. Consequently, during our synthesis, both phases can form competitively, and the transformation toward the thermodynamically favored phase can be kinetically slow, requiring a long reaction time. Therefore, distinct from prior short-duration syntheses, we believe our results point toward “equilibrium-like” conditions for achieving metastable γ–TaON, and this is the subject of ongoing research by our team.

#### 3.3.3 Probing Validated β-TaON Phase Diagram with Coflown N_2_ Gas

The consistent prevalence of secondary phases in the predominant β–TaON products synthesized via ammonolysis under the flow of NH_3_, H_2_O delivered via Ar, and H_2_ warranted close consideration of synthetic parameters, especially gas species. NH_3_ and H_2_O are respective sources of nitrogen and oxygen in the reaction, and H_2_ is not assumed to be actively involved in the reactions from Ta_2_O_5_ to TaON and Ta_2_O_5_ to Ta_3_N_5_ (see eqs 1 and 2, respectively). However, H_2_ is a known reducing agent, which raises the question of its role in the ammonolysis reaction. To probe the impact of H_2_ on product phases, the same ammonolysis reactions as those discussed in Section 3.3.1 and 3.3.2were repeated, but with N_2_ coflown instead of H_2_ gas. As such, the partial pressures of NH_3_ and H_2_O keep invariant. The reaction temperatures and gas flow rates are shown in Table 1. As the Ellingham-type phase diagrams are plotted as a function of reactive gas partial pressures, the exact points could be replicated. The predicted phase diagram and phase equilibria lines remained unchanged, such that the additional experimental trials served as further validation of their accuracy. Products from ammonolysis reactions at both 1050 and 1100 K using coflown N_2_ gas are shown in Figure 5. As seen in Figure 5a and 5d, there is good agreement between experiment and computation across reaction temperatures; the products that are predominantly β–TaON fall within the predicted synthetic window, and the two-phase mixtures appear quite close to the predicted phase boundaries. The extracted phase fractions of products from Rietveld refinement are shown in Figure 5b and 5c for 1050 K (see Figures S10 and S11 for measured and calculated XRD patterns) and Figure 5e and 5f for 1100 K (see Figures S12 and S13 for measured and calculated XRD patterns). Interestingly, the phase purities of the β–TaON products (>99%) are vastly improved compared to the predominant β–TaON products synthesized with coflown H_2_. This finding points to H_2_ playing a minor but significant role in the ammonolysis reaction, possibly by acting as a reducing agent and reducing the surface of the bulk powders. Further investigation of the powder surface and bulk structure is needed to determine the precise contribution of H_2_.

**Figure 5:**
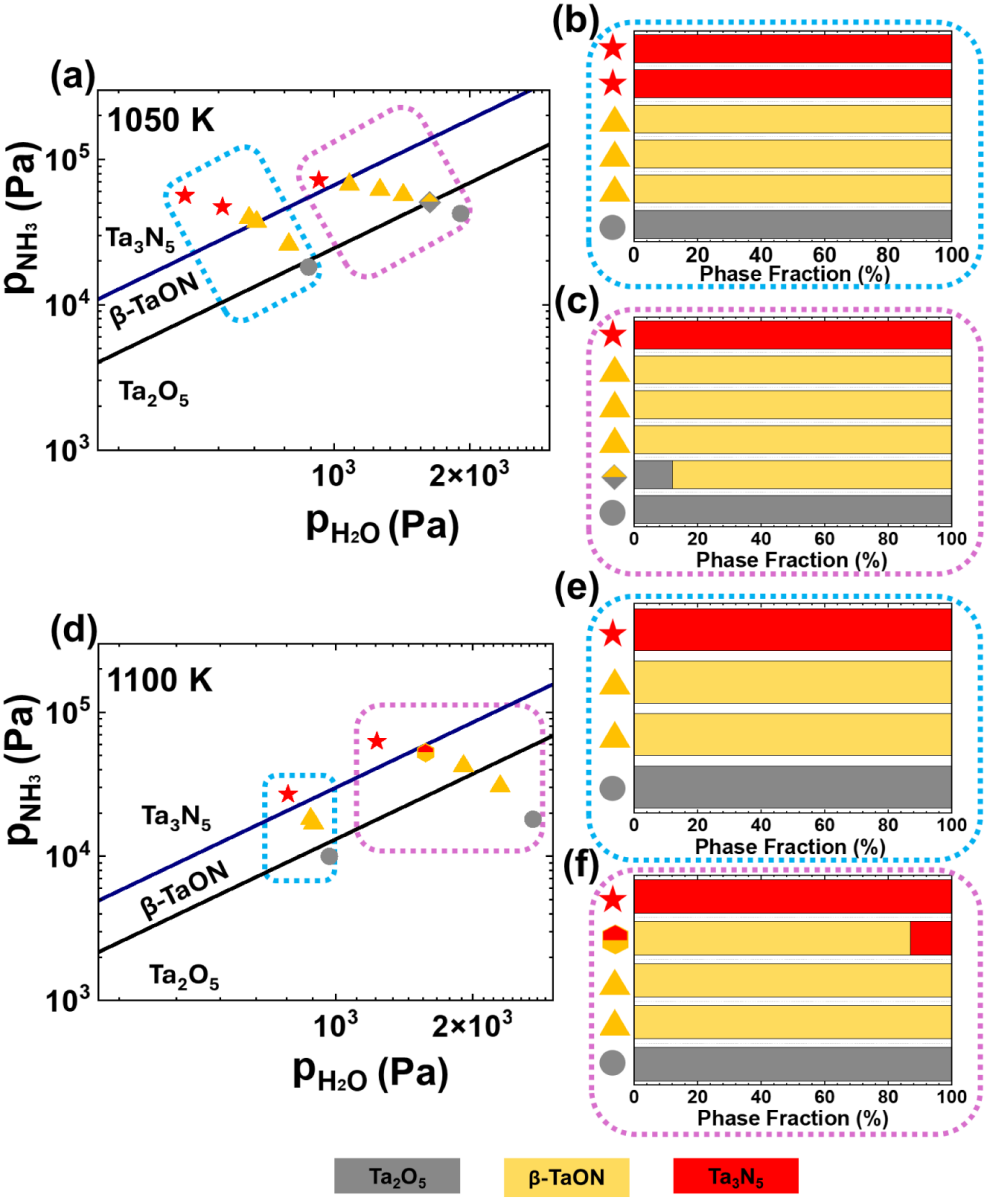
(a) Computationally predicted Ellingham-type diagram of β-TaON synthesis window at 1050 K with experimental synthetic validation (combined gas flow of Ar/H_2_O, N_2_ and NH_3_). (b),(c) Phase fractions of products (1050 K reaction temperature) at lower and higher water flow rates, respectively. (d) Computationally predicted Ellingham-type diagram of β-TaON synthesis window at 1100 K with a combined gas flow of Ar/H_2_O, N_2_ and NH_3_ with experimental synthetic validation. (e),(f) Phase fractions of products (1100 K reaction temperature) at lower and higher water flow rates, respectively. In (a) and (d), black and blue lines correspond to the Ta_2_O_5_/β–TaON and Ta_3_N_5_/β–TaON phase boundaries, respectively. Gray circles, yellow triangles, and red five-point stars denote majority (>90%) Ta_2_O_5_, TaON, Ta_3_N_5_ product phases, respectively. Dual-colored markers indicate the conditions yielding products containing two phases with significant amounts, where the gray/yellow dual-colored four-point diamond shapes denote Ta_2_O_5_/TaON two-phase products, while the red/yellow dual-colored hexagons denote a TaON/Ta_3_N_5_ mixed product. In (b), (c), (e), and (f), red, yellow, and gray colors correspond to Ta_3_N_5_, β-TaON, and Ta_2_O_5_, respectively, and the symbols from bottom to top in each plot are in order of increasing partial pressure of NH_3_ while keeping water flow rate constant.

#### 3.3.4 Probing Validated β-TaON Phase Diagram without Coflown Gases

It is clear that coflown gases (e.g., H_2_, N_2_) can be impactful on the final ammonolysis products, as noted with the higher phase purity β–TaON products while coflowing N_2_ as compared to H_2_. However, it was also observed experimentally that, regardless of coflown gases, phase boundaries between Ta_2_O_5_/β–TaON and Ta_3_N_5_/β–TaON remain fixed, underscoring the foundational connection between $p_{{\mathrm{NH}}_{3}}$ and $p_{H_{2}O}$ and reaction thermodynamics. To further validate the predicted β-TaON synthetic window, ammonolysis reactions at 1050 and 1100 K were performed during which only NH_3_ and H_2_O (delivered via Ar) were flowed (Table 1). Points on the phase diagram corresponding to specific pairs of $p_{{\mathrm{NH}}_{3}}$ and $p_{H_{2}O}$ values were chosen such that the phase boundaries and β-TaON window could be probed. As seen in Figure 6a and 6b at 1050 K and Figure 6c and 6d at 1100 K, the products arising from ammonolysis reactions match well with the predicted phase diagram: predominant β-TaON products appear in the predicted synthetic window and two-phase mixtures appear near or on the predicted phase boundaries. Unique to these synthesis trials without coflown N_2_ or H_2_ gases is the role kinetics seems to be playing in the formation of β-TaON as a product. As shown via extracted phase fractions of products in Figure 6b at 1050 K (see Figure S14 for measured and calculated XRD patterns) and Figure 6d at 1100 K (see Figure S15 for measured and calculated XRD patterns), the transformation to β-TaON requires longer reaction hold times (>40 h) than in previous ammonolysis trials (20 h). Clearly, the transformation to equilibrium β-TaON is favored over longer holds, as seen in the growing phase fraction of β-TaON as compared to Ta_2_O_5_ in the 40 h hold as compared to the 20 h hold, which suggests that kinetics is the main driver of the mixed-phase product formation without N_2_ or H_2_ coflown gases. Thus, while the addition of coflown gases does not shift the phase boundaries, it does appear to impact the ammonolysis reaction kinetics.

**Figure 6:**
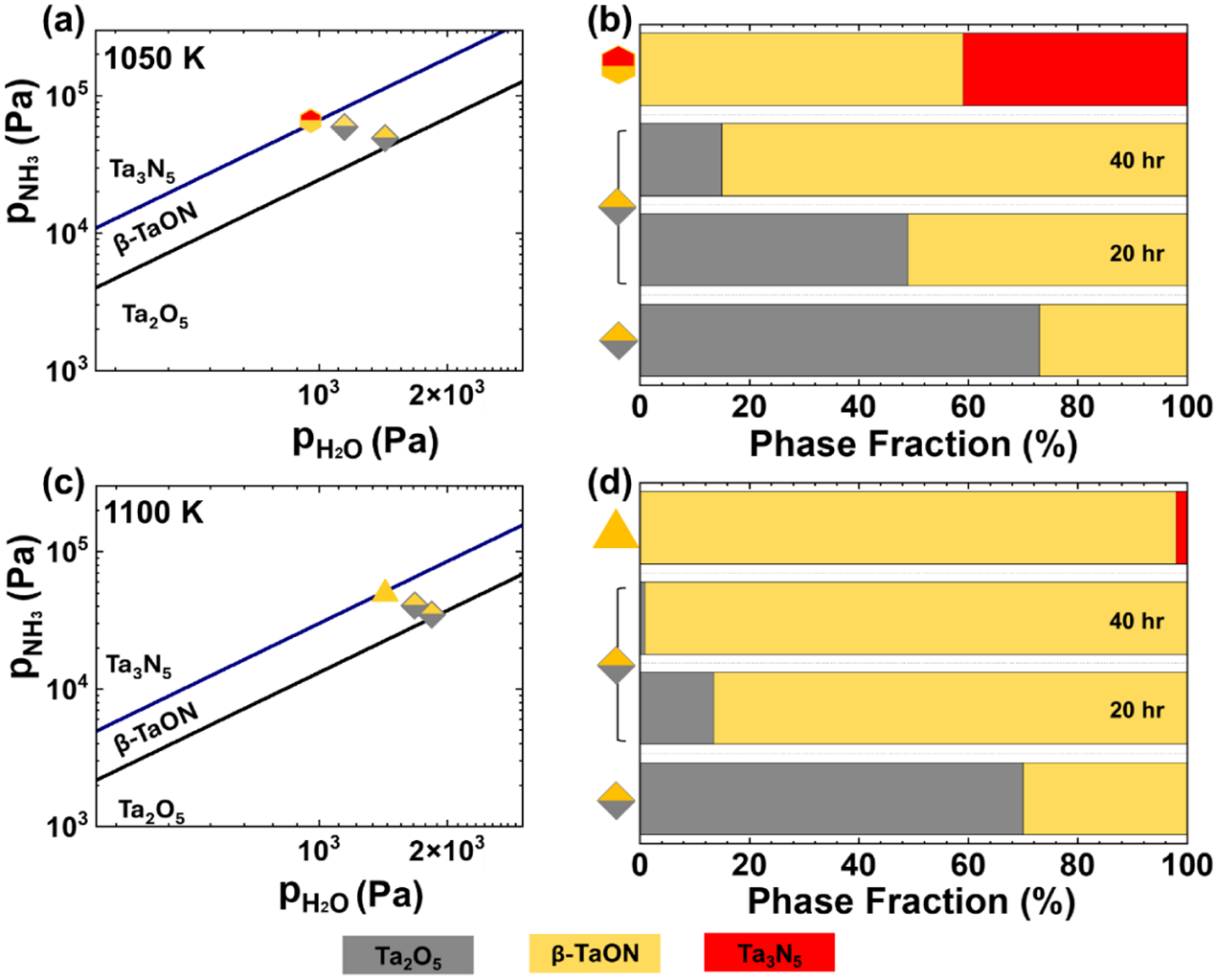
(a) Computationally predicted Ellingham-type diagram of β-TaON synthesis window at 1050 K with experimental synthetic validation (combined gas flow of Ar/H_2_O and NH_3_). (b) Phase fractions of products (1050 K reaction temperature), including repeated trial with 40 h reaction time. (c) Computationally predicted Ellingham diagram-type of β-TaON synthesis window at 1100 K with a combined gas flow of Ar/H_2_O and NH_3_ with experimental synthetic validation. (d) Phase fractions of products (1100 K reaction temperature), including a repeated trial with 40 h reaction time. In (a) and (c), black and blue lines correspond to the Ta_2_O_5_/β–TaON and Ta_3_N_5_/β–TaON phase boundaries, respectively. Gray circles, yellow triangles, and red five-point stars denote majority (>90%) Ta_2_O_5_, TaON, Ta_3_N_5_ product phases, respectively. Dual-colored markers indicate the conditions yielding products containing two phases in significant amounts, where the gray/yellow dual-colored four-point diamond shapes denote Ta_2_O_5_/TaON two-phase products, while the red/yellow dual-colored hexagons denote a TaON/Ta_3_N_5_ mixed product. In (b) and (d), red, yellow, and gray colors correspond to Ta_3_N_5_, β-TaON, and Ta_2_O_5_, respectively and the symbols from bottom to top in each plot are in order of increasing partial pressure of NH_3_ while keeping water flow rate constant.

#### 3.3.5 Synthesis Window of β-TaON in Three Dimensions

Using the validated CALPHAD model at 1050 and 1100 K across different gas flow scenarios, we predict a 3D phase diagram that reveals the coupled effects of temperature and partial pressures of NH_3_ and H_2_O on the synthesis window (i.e., single-phase region) of β-TaON. As shown in Figure 7, across a wide range of temperatures, β-TaON maintains a very narrow synthesis window with respect to $p_{{\mathrm{NH}}_{3}}$ and $p_{H_{2}O}$ . This likely explains why the phase-pure synthesis of β-TaON at equilibrium conditions has been historically challenging and difficult to reproduce, as it requires stringent control of gas flow rates and their ratios within a narrow range. We note that such a narrow synthesis window of β-TaON reflects an intrinsic thermodynamic feature of the Ta–O–N system, rather than kinetic constraints of the synthesis reaction. A narrow single-phase region of β-TaON was also reported by Chen et al. in their work on 0 K DFT-computed chemical potential phase diagrams.^77^ They showed that β-TaON occupies a narrow stability region with respect to the chemical potential of oxygen and nitrogen, bounded by Ta_2_O_5_ at lower *μ*_*N*_ and Ta_3_N_5_ at higher *μ_N_*. The agreement between their reported results and the observations in the present work lends further support to a thermodynamic origin of the narrow synthesis window.

**Figure 7:**
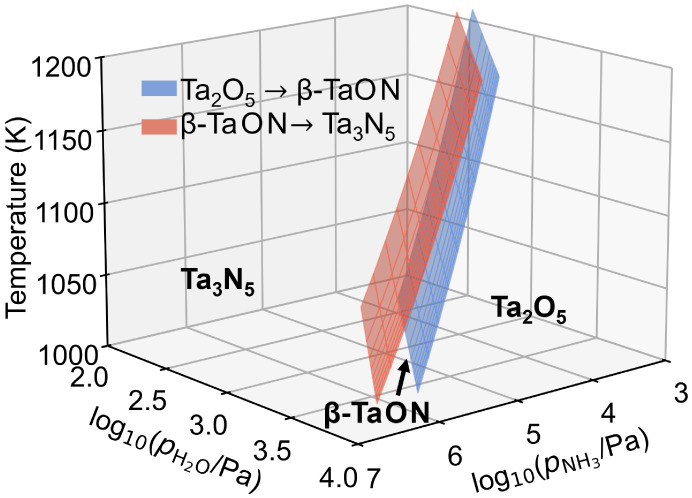
Three-dimensional synthesis window of β-TaON as a function of temperature and reactive gas partial pressures, constructed from the finalized CALPHAD model. The blue surface represents the equilibrium phase boundary between Ta_2_O_5_ and β-TaON, while the red surface corresponds to the phase boundary between β-TaON and Ta_3_N_5_. The partial pressures of NH_3_ and H_2_O are plotted on a logarithmic scale.

In addition to partial pressures, temperature also exerts a profound influence on the β-TaON synthesis window. Figure 7 clearly shows that the single-phase region of β-TaON becomes narrower as temperature increases, suggesting that high ammonolysis temperature can easily lead to overnitridation of Ta_2_O_5_ to Ta_3_N_5_, unless the gas flow conditions are carefully tuned (see Figure S16 for the 1200 K Ellingham-type diagram with experimental validation), despite the benefit of accelerated reaction kinetics afforded by the high temperature. More importantly, although most reported syntheses of β-TaON have been conducted around 1100 K, likely influenced by seminal works from Brauer and Weidlein^38^ and Swisher and Read,^30^ our experimental and modeling results demonstrate that slightly lower ammonolysis temperatures are beneficial for the synthesis of β-TaON as it broadens the synthesis window. However, it should also be acknowledged that ammonolysis temperatures that are too low may kinetically hinder or even suppress the ammonolysis reaction. Additional ammonolysis studies completed at 950 and 1000 K within the predicted β-TaON window yielded mixed-phase Ta_2_O_5_/Ta_3_N_5_ and majority β-TaON (>70%), respectively, as products (see Figures S17 and S18). These results are attributed to slower reaction kinetics at lower temperatures that limit the extent of ammonolysis (i.e., reaction results at 1000 K) and to the requirement of higher NH_3_ partial pressures during ammonolysis that may cause strong local fluctuations in the NH_3_ chemical potential, leading to over nitridation to form Ta_3_N_5_ (i.e., reaction results at 950 K). This warrants a future study on the reaction kinetics and the temporal evolution of the phase diagram predicted in the present work to provide a well-considered determination of the optimal synthesis temperature for β-TaON.

## 4 Conclusions

In this work, an integrated computational-experimental framework was developed to understand and predict the equilibrium synthesis window of β-TaON, a representative mixed-anion compound characterized by complex phase behavior, as a function of temperature and gas flow rates, which are key control parameters in ammonolysis-based syntheses. By integrating first-principles QHA calculations with CALPHAD modeling, we constructed temperature-dependent Gibbs free energy functions for β-TaON and Ta_3_N_5_, enabling rigorous mapping of phase equilibria as a function of synthesis temperature and reactive gas partial pressures. Based on the ideal-gas assumption, these partial pressures can be quantitatively related to the volumetric flow rates of the individual gas species—parameters that can be directly controlled in our synthesis experiments.

Ammonolysis reactions carried out across a range of gas conditions and temperatures validated the predicted phase boundaries and confirmed the presence of a narrow synthesis window for β-TaON. Iterative refinement of the CALPHAD model using experimental phase assemblages enabled quantitative correction of β-TaON and Ta_3_N_5_ Gibbs free energies, aligning theoretical predictions with observed equilibria while preserving fidelity to first-principles energetics. The breadth of the synthesis window of β-TaON was found to be highly sensitive to both reactive gas partial pressures and reaction temperature, with a lower reaction temperature generally broadening the synthesis window. Furthermore, co-flown gases in addition to NH_3_ and H_2_O (delivered via Ar) played important roles in the final products: the addition of N_2_ led to greater phase purity of β-TaON products and improved the kinetics of the reaction as compared to coflowing H_2_ or flowing no additional gases, respectively.

This work reveals the limitations of prior empirical synthesis maps and underscores the need for thermodynamically grounded modeling coupled with experimental validation. The integrated DFT-CALPHAD-experimental framework presented here offers a strategy for mapping the complex synthesis window for β-TaON. Beyond mixed-anion materials, this approach provides a blueprint for the rational design of synthesis conditions for functional inorganic material systems with complex chemistries broadly.

## Supplementary Material



## Notes

The accompanying SI contains DFT-predicted phonon dispersion and density of states for β-TaON at 1.06x its equilibrium volume, reproduced *G*(*T*) curve of Ta_2_O_5_ expressed in the CALPHAD reference states based on DFT-QHA calculations, refined XRD patterns for synthetic trials discussed in the manuscript, product phase fractions for extended reaction time trials with coflown H_2_, phase boundaries predicted as a function of $\frac{y_{H_{2}O}}{y_{H_{2}}}$ and $\frac{y_{{\mathrm{NH}}_{3}}}{y_{H_{2}}^{3/2}}$ and experimental synthetic validation for reactions with coflown H_2_ at 1100 K, Ellingham-type diagrams of β-TaON synthesis window at 950, 1000, and 1200 K, and corresponding XRD patterns of products for experimental validation, XRD patterns of products formed during dry and wet ammonolysis with different maximum reaction temperature hold times, table of benchmark of DFT-computed ZPE and thermal corrections for N_2_ and O_2_ gas molecules, and table of formation enthalpy of Ta-O-N compounds calculated using different methods (PDF)
