# Supplementary material for: Establishing the Temperature-Dependent Synthesis Window of β‑TaON by Coupling Predictive Thermodynamic Modeling with Experimental Validation
Source: Chem Mater. 2026 Jun 29;38(13):6287–301. doi: 10.1021/acs.chemmater.6c00139 (PMC13373923; doi:10.1021/acs.chemmater.6c00139)
Supplement: Supplementary file 1 [file cm6c00139_si_001.pdf]

Supplementary Information

Establishing the temperature-dependent synthesis window of β-TaON by coupling predictive thermodynamic modeling with experimental validation

Aksha Gilbert Prince[ǂ], Yuanchen Gao[ǂ], Dmitri LaBelle, Jill K. Wenderott* and Yong-Jie Hu*

Department of Materials Science and Engineering, Drexel University, Philadelphia, PA 19104, United States

[ǂ] Contributed equally to this work
*Corresponding authors: jw3866@drexel.edu; yh593@drexel.edu

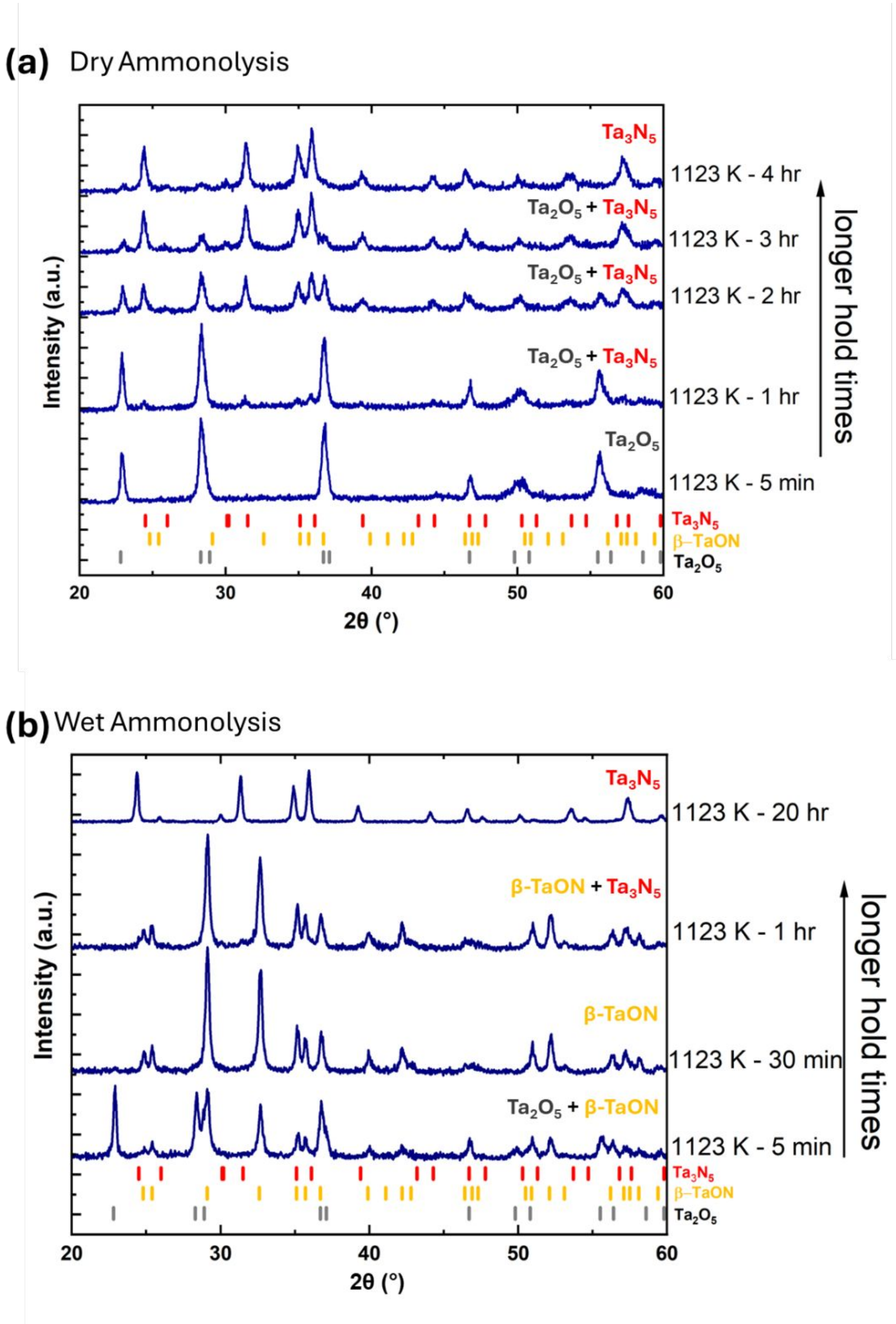

Figure S1. (a) Dry ammonolysis with varying maximum reaction temperature hold times during which the direct evolution of Ta_3N_5 from Ta_2O_5 without β-TaON as an intermediate is observed. The reaction was carried out under 100 sccm of dry NH_3 gas. (b) Wet ammonolysis with varying maximum reaction temperature hold times during which the transition of Ta_2O_5 to Ta_3N_5 through β-TaON as an intermediate is observed. The reaction was carried out under 100 sccm of NH_3 gas along with 60 sccm of humidified Ar gas (corresponding to 1.2 sccm of H_2O).

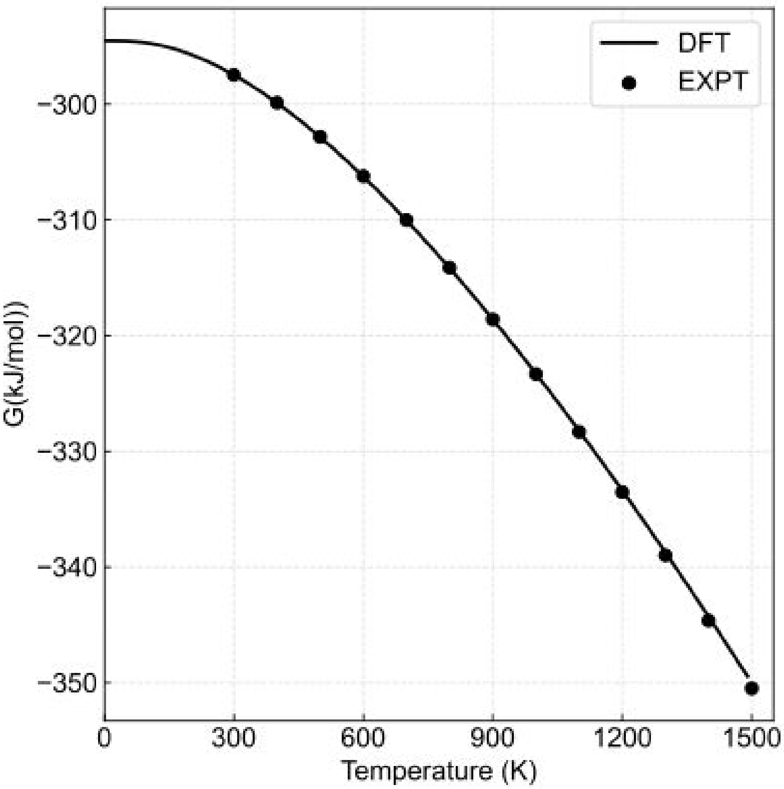

Figure S2 Reproduced G(T) curve of Ta_2O_5 expressed in the CALPHAD reference states, with parameters determined from thermodynamic properties predicted by DFT-QHA calculations, compared with data points (EXPT) obtained from literature based on experimental measurements[1,2] .

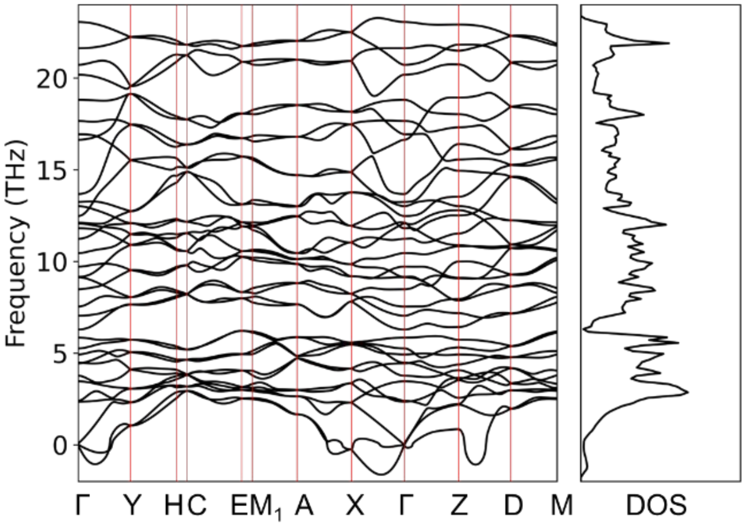

Figure S3. DFT-predicted phonon dispersion and density of states for β-TaON at 1.06 times of its equilibrium volume at 0K.

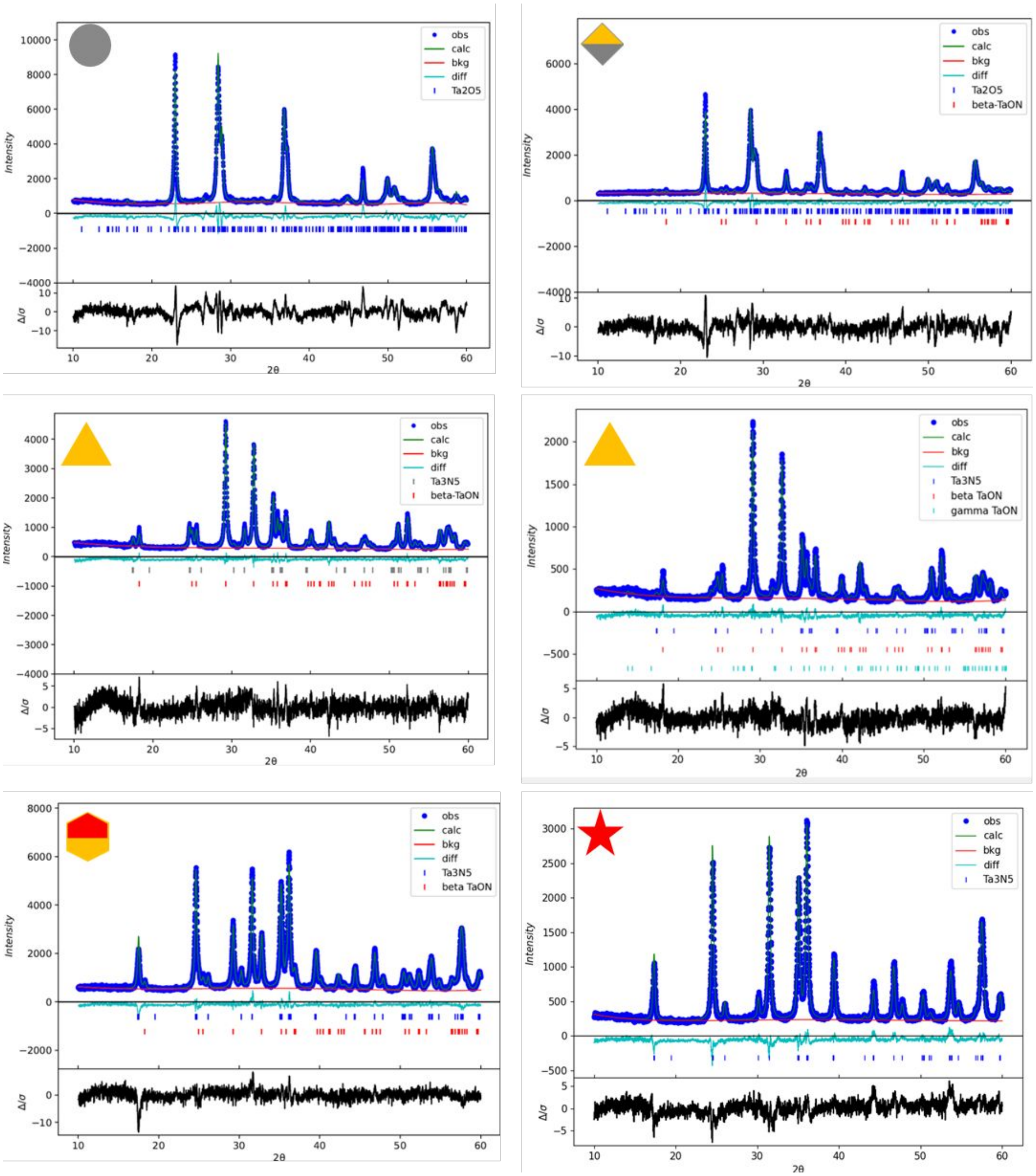

Figure S4. Measured (blue dots) and calculated (green line) XRD diffraction data, the latter from Rietveld refinement, for 1050 K reaction temperature products at lower water partial pressures (gas mixture containing NH_3, H_2O via Ar, and H_2). Symbols correspond to the respective products in the phase diagram (moving from lower to higher ammonia partial pressures). Refer to **Figure 4b** in main manuscript.

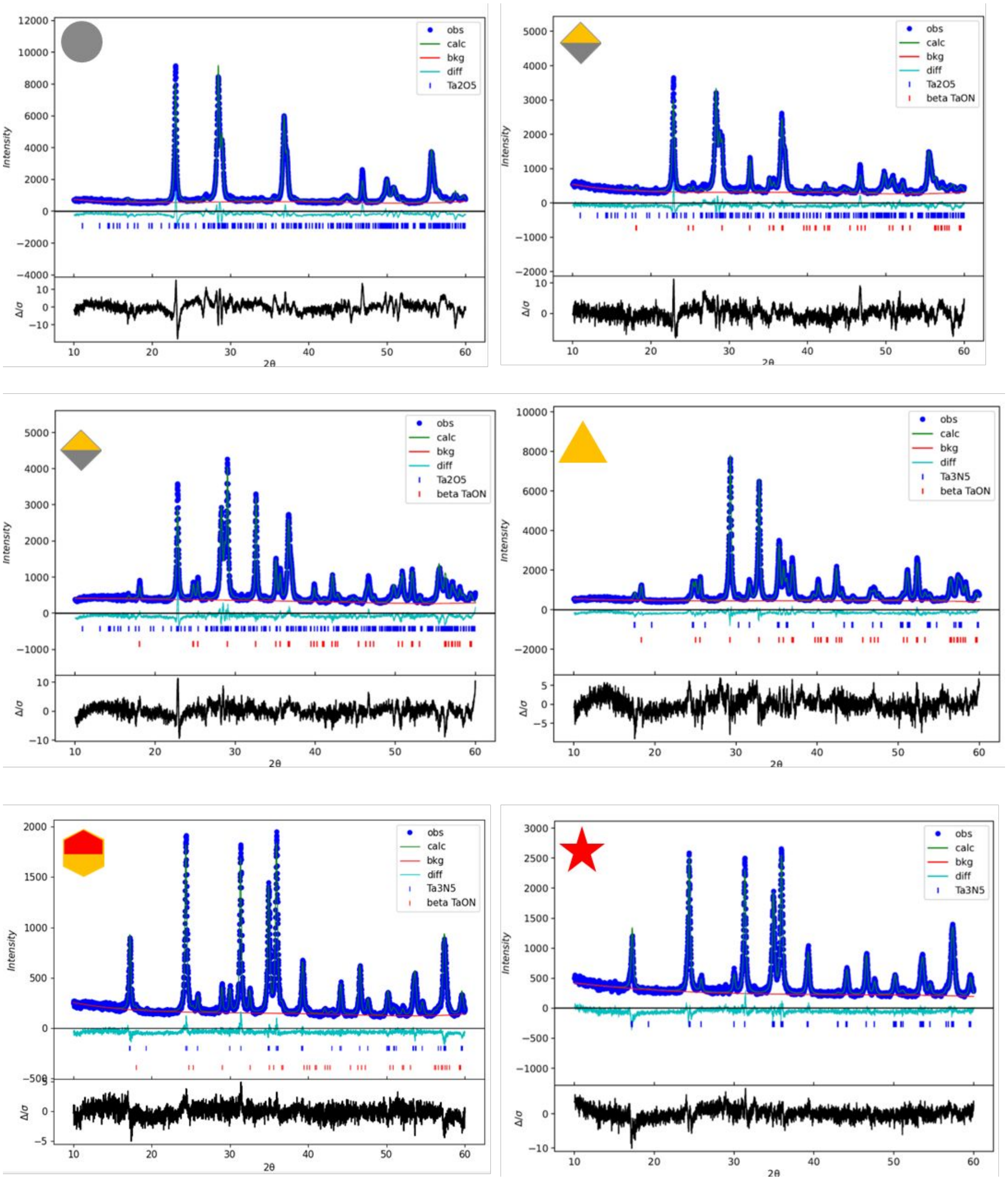

Figure S5. Measured (blue dots) and calculated (green line) XRD diffraction data, the latter from Rietveld refinement, for 1050 K reaction temperature products at higher water partial pressures (gas mixture containing NH_3, H_2O via Ar, and H_2). Symbols correspond to the respective products in the phase diagram (moving from lower to higher ammonia partial pressures). Refer to **Figure 4c** in main manuscript.

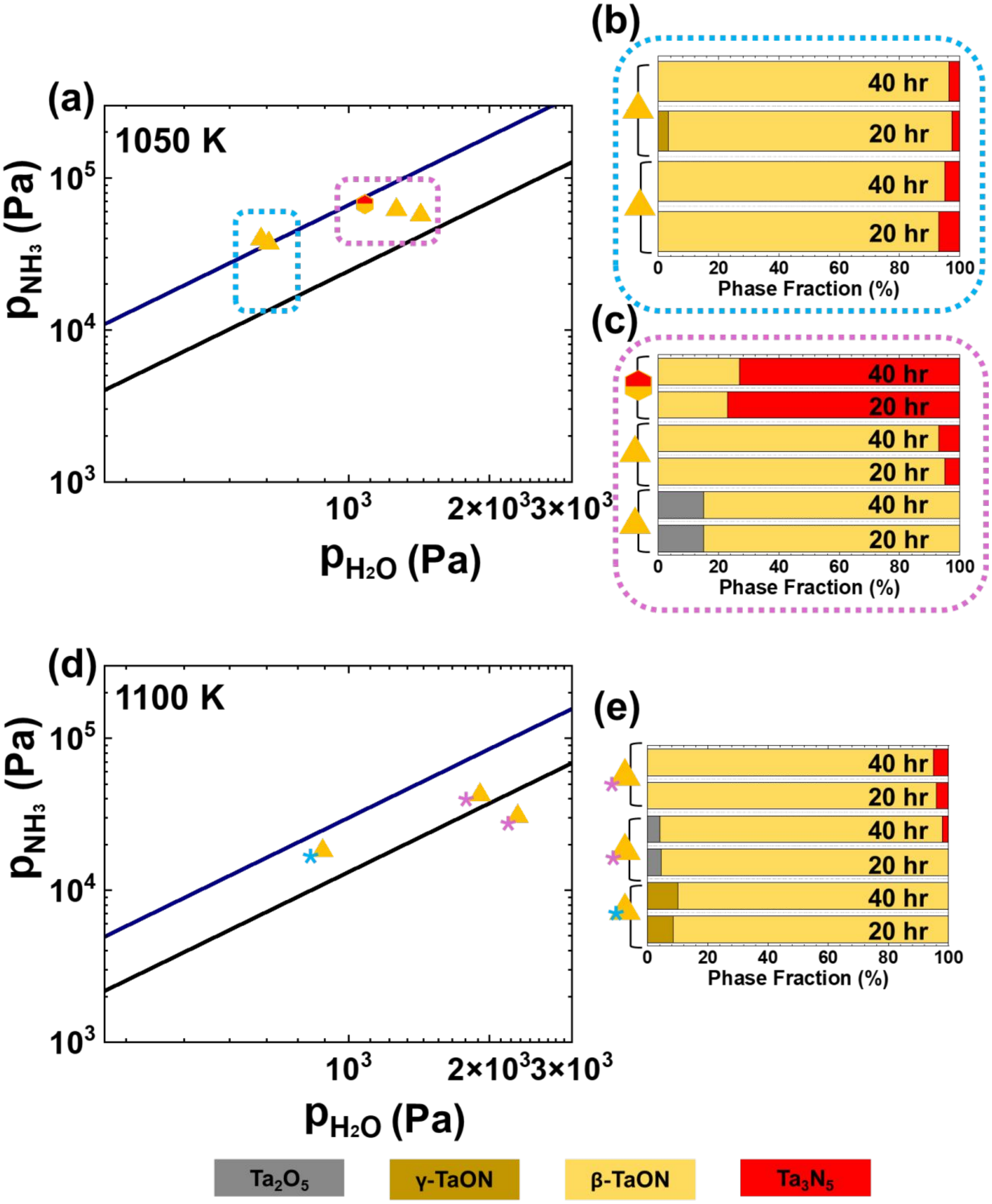

Figure S6. Comparison of product phase fractions for 20 hr and 40 hr reaction times at 1050 K and 1100 K with gas mixture of NH_3, H_2O via Ar, and H_2. In (a) and (d), black and blue lines correspond to the Ta_2O_5/β–TaON and Ta_3N_5/β–TaON phase boundaries, respectively. Yellow triangles and red five-point stars denote majority (>90%) TaON and Ta_3N_5 product phases, respectively. In (b), (c) and (e), red, yellow, and grey colors correspond to Ta_3N_5, β-TaON, and Ta_2O_5, respectively and the symbols from bottom to top in each plot are in order of increasing the partial pressure of NH_3 while keeping the flow rate of water as constant.

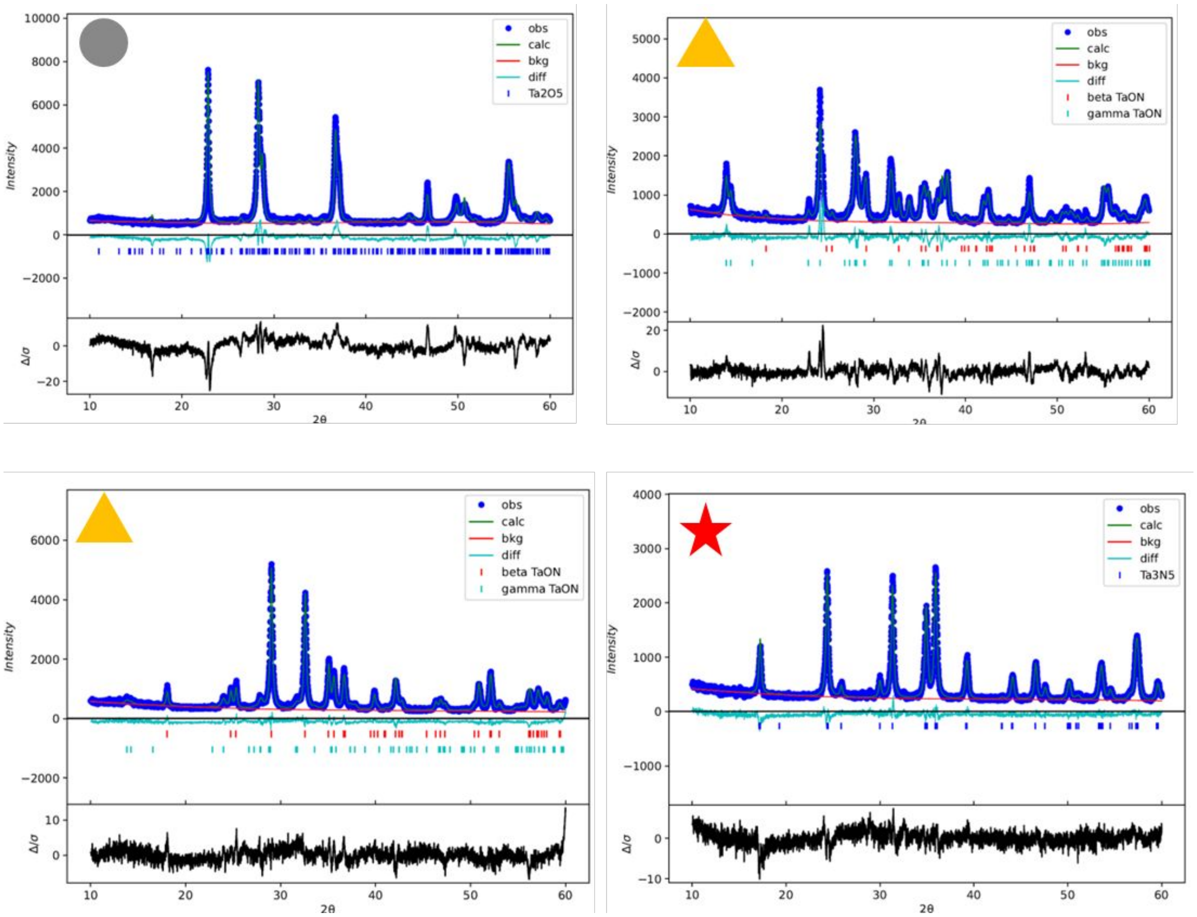

Figure S7. Measured (blue dots) and calculated (green line) XRD diffraction data, the latter from Rietveld refinement, for 1100 K reaction temperature products at lower water partial pressures (gas mixture containing NH_3, H_2O via Ar, and H_2). Symbols correspond to the respective products in the phase diagram (moving from lower to higher ammonia partial pressures). Refer to **Figure 4e** in main manuscript.

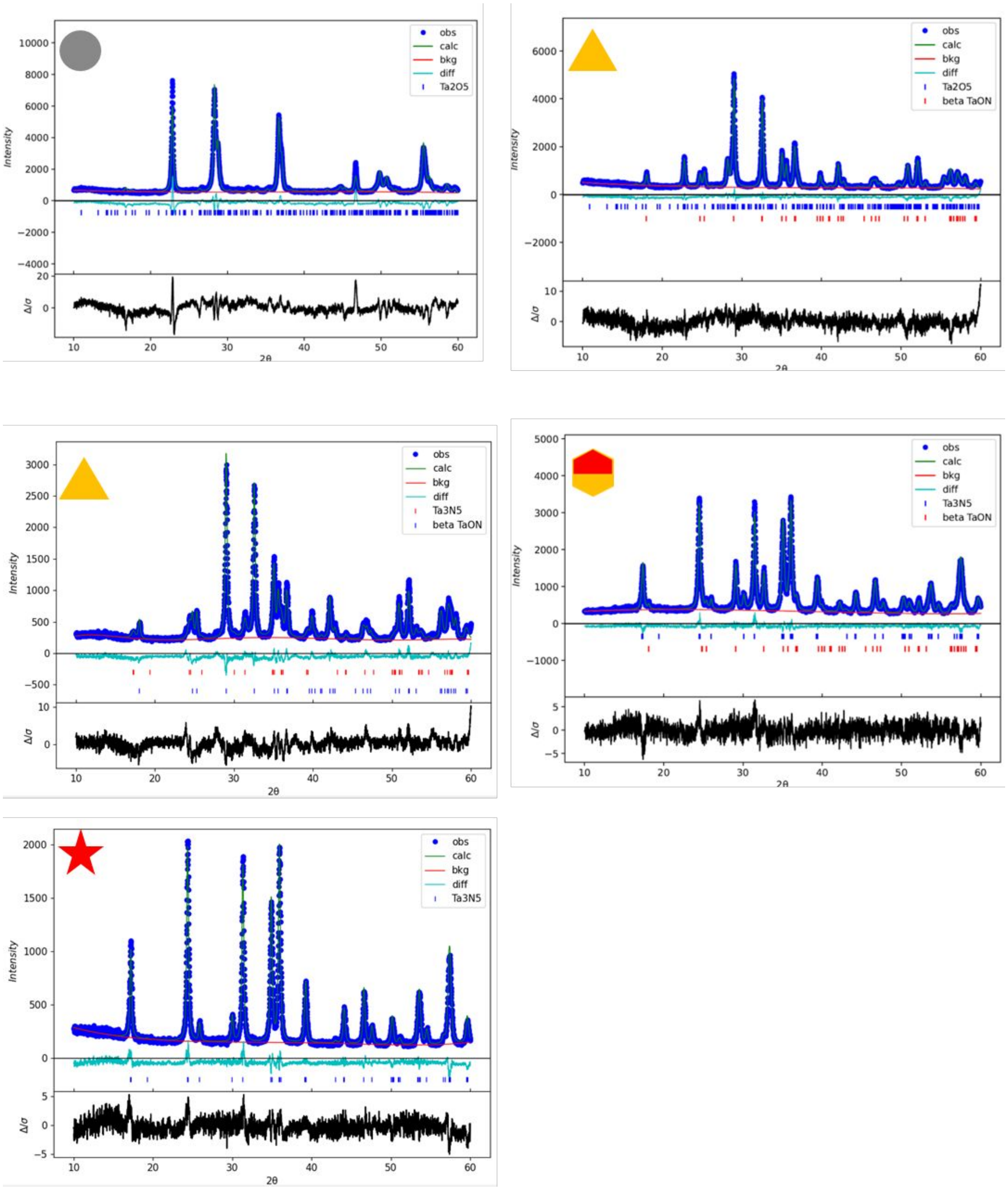

Figure S8. Measured (blue dots) and calculated (green line) XRD diffraction data, the latter from Rietveld refinement, for 1100 K reaction temperature products at higher water partial pressures (gas mixture containing NH_3, H_2O via Ar, and H_2). Symbols correspond to the respective products in the phase diagram (moving from lower to higher ammonia partial pressures). Refer to **Figure 4f** in main manuscript.

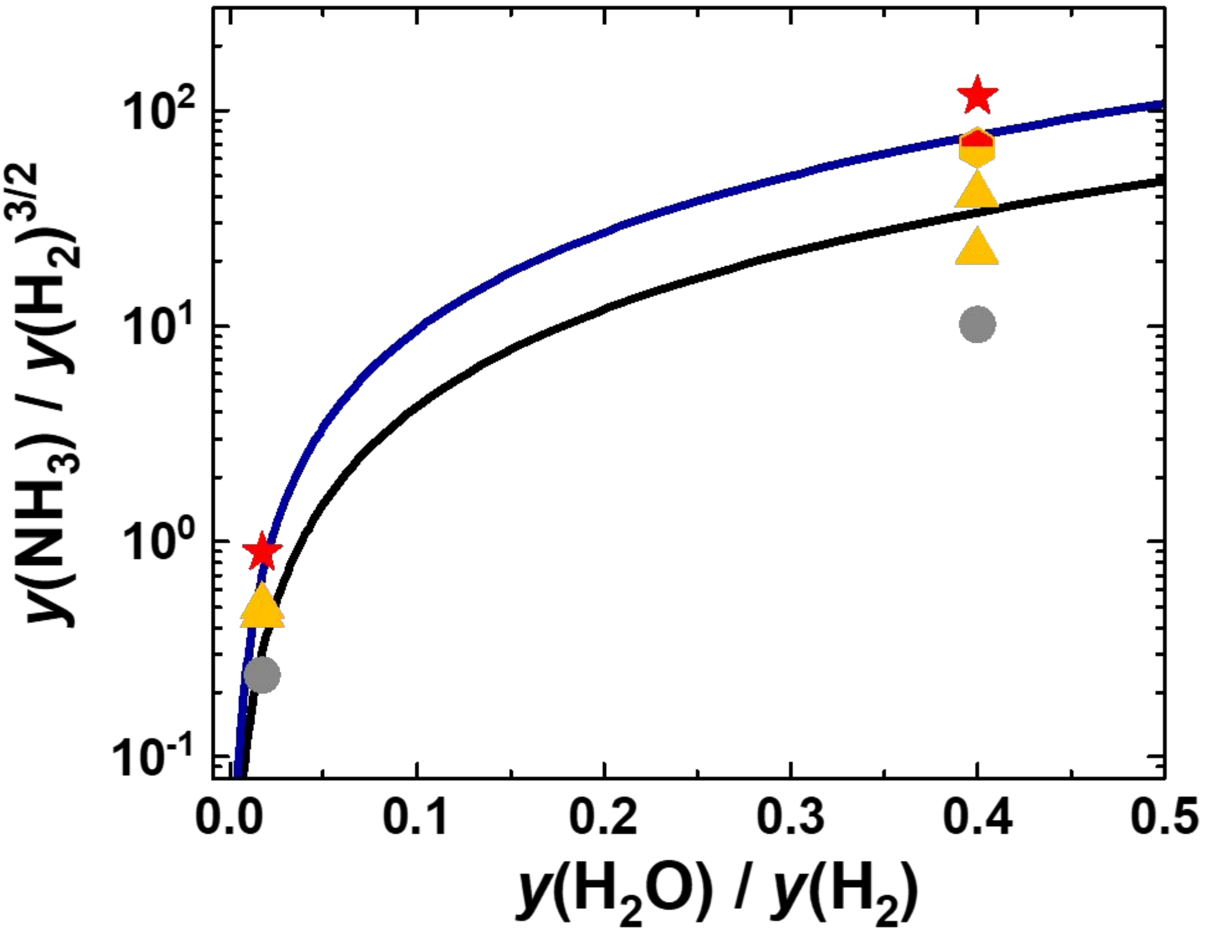

Figure S9. Computationally-predicted phase diagram of β–TaON at 1100 K with experimental synthetic validation (combined gas flow of Ar/H_2O, H_2, and NH_3). The black and blue lines correspond to the oxide/oxynitride and nitride/oxynitride transition lines, respectively. Phase fractions of products at lower and higher water flow rates are found in **Figure 4e** and **4f** in main manuscript, respectively.

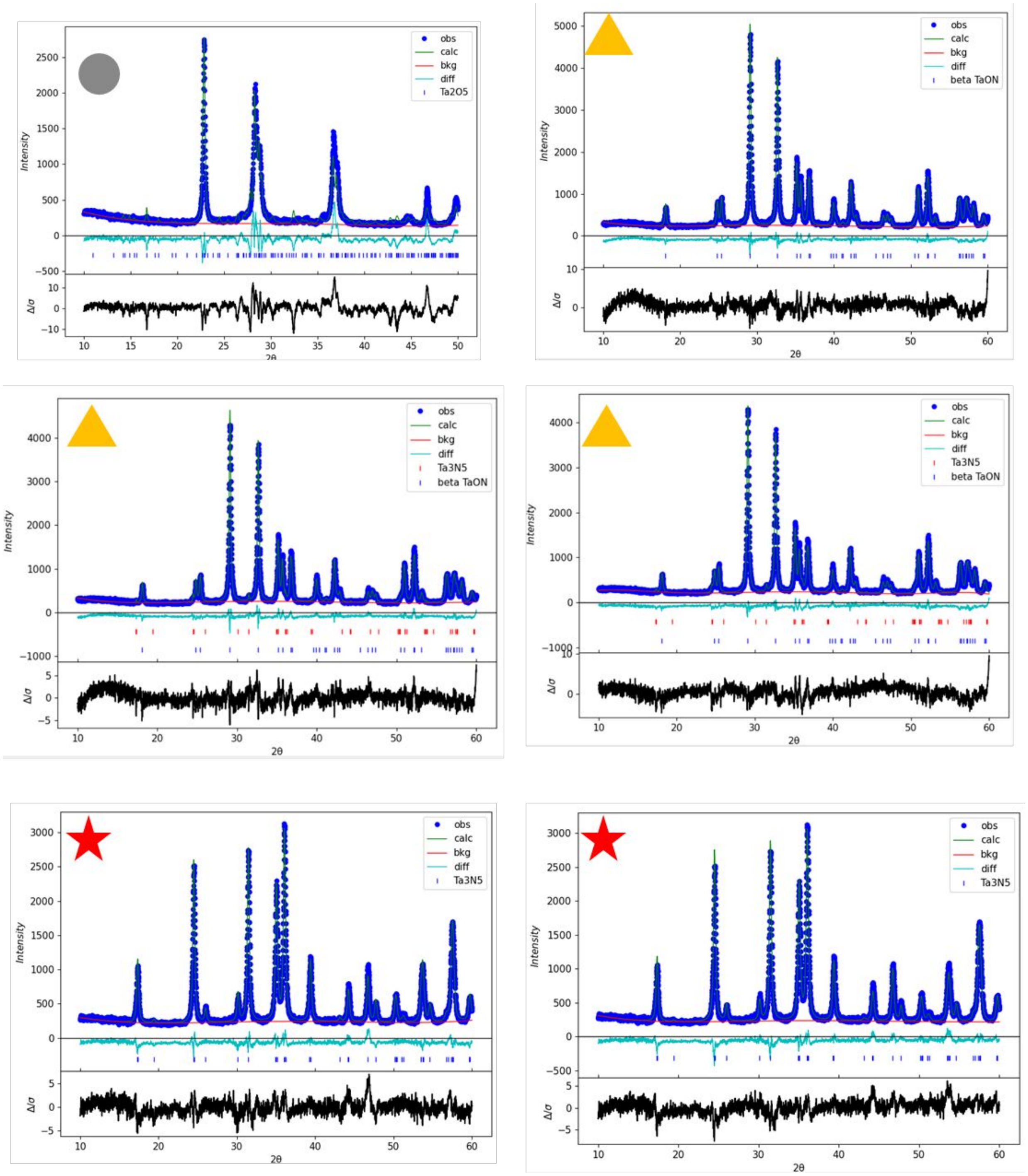

Figure S10. Measured (blue dots) and calculated (green line) XRD diffraction data, the latter from Rietveld refinement, for 1050 K reaction temperature products at lower water partial pressures (gas mixture containing NH_3, H_2O via Ar, and N_2). Symbols correspond to the respective products in the phase diagram (moving from lower to higher ammonia partial pressures). Refer to **Figure 5b** in main manuscript.

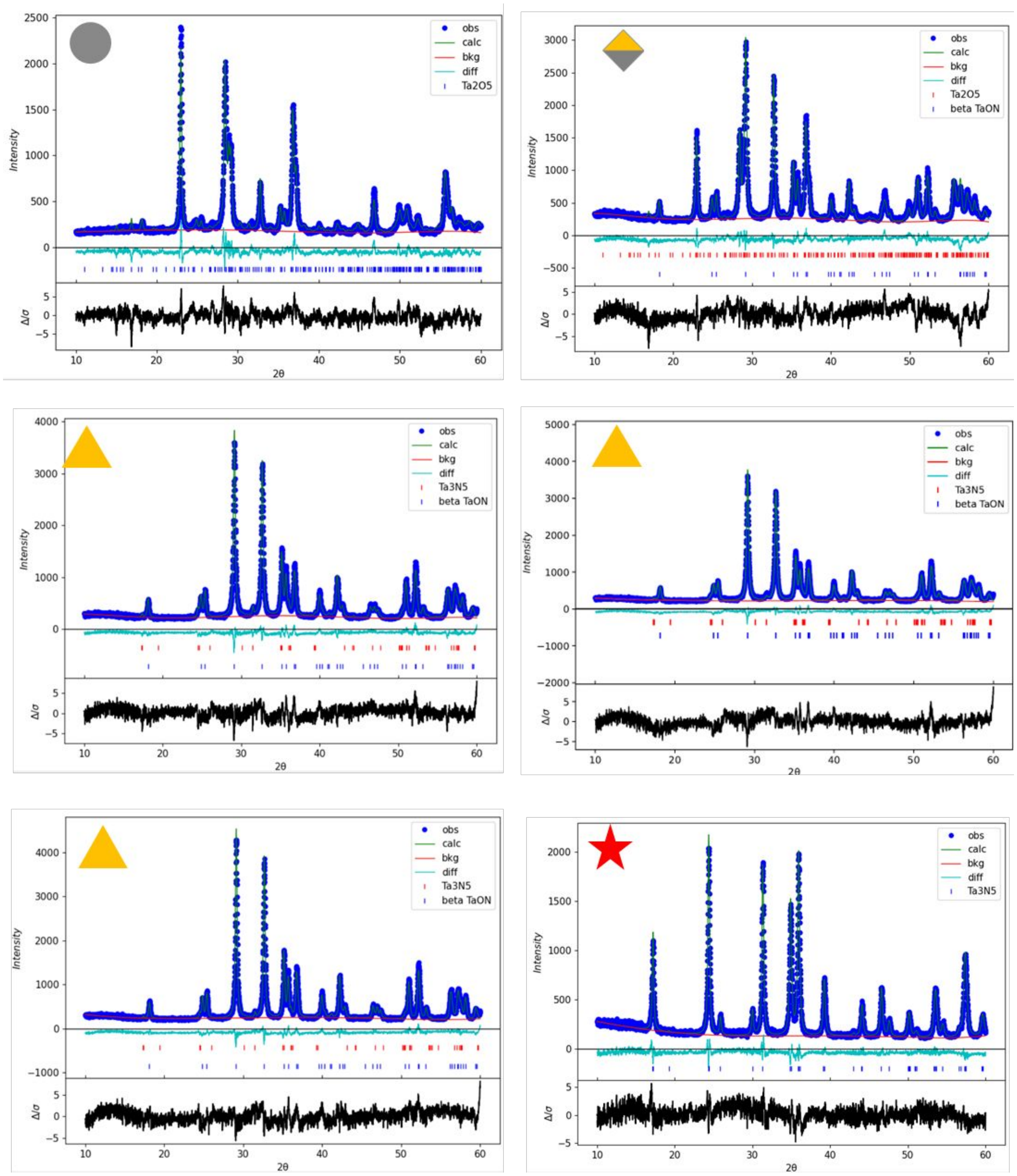

Figure S11. Measured (blue dots) and calculated (green line) XRD diffraction data, the latter from Rietveld refinement, for 1050 K reaction temperature products at higher water partial pressures (gas mixture containing NH_3, H_2O via Ar, and N_2). Symbols correspond to the respective products in the phase diagram (moving from lower to higher ammonia partial pressures). Refer to **Figure 5c** in main manuscript.

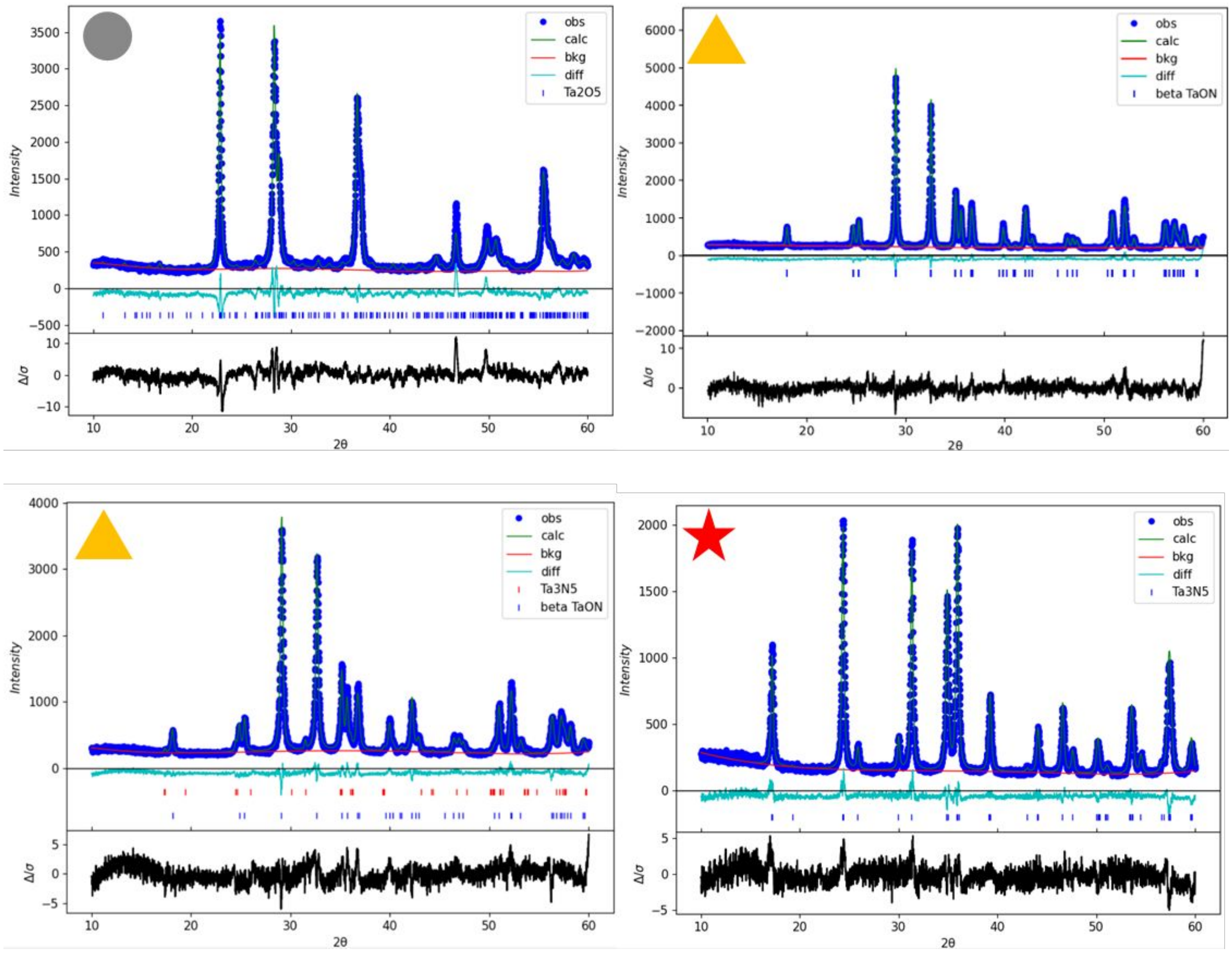

Figure S12. Measured (blue dots) and calculated (green line) XRD diffraction data, the latter from Rietveld refinement, for 1100 K reaction temperature products at lower water partial pressures (gas mixture containing NH_3, H_2O via Ar, and N_2). Symbols correspond to the respective products in the phase diagram (moving from lower to higher ammonia partial pressures). Refer to **Figure 5e** in main manuscript.

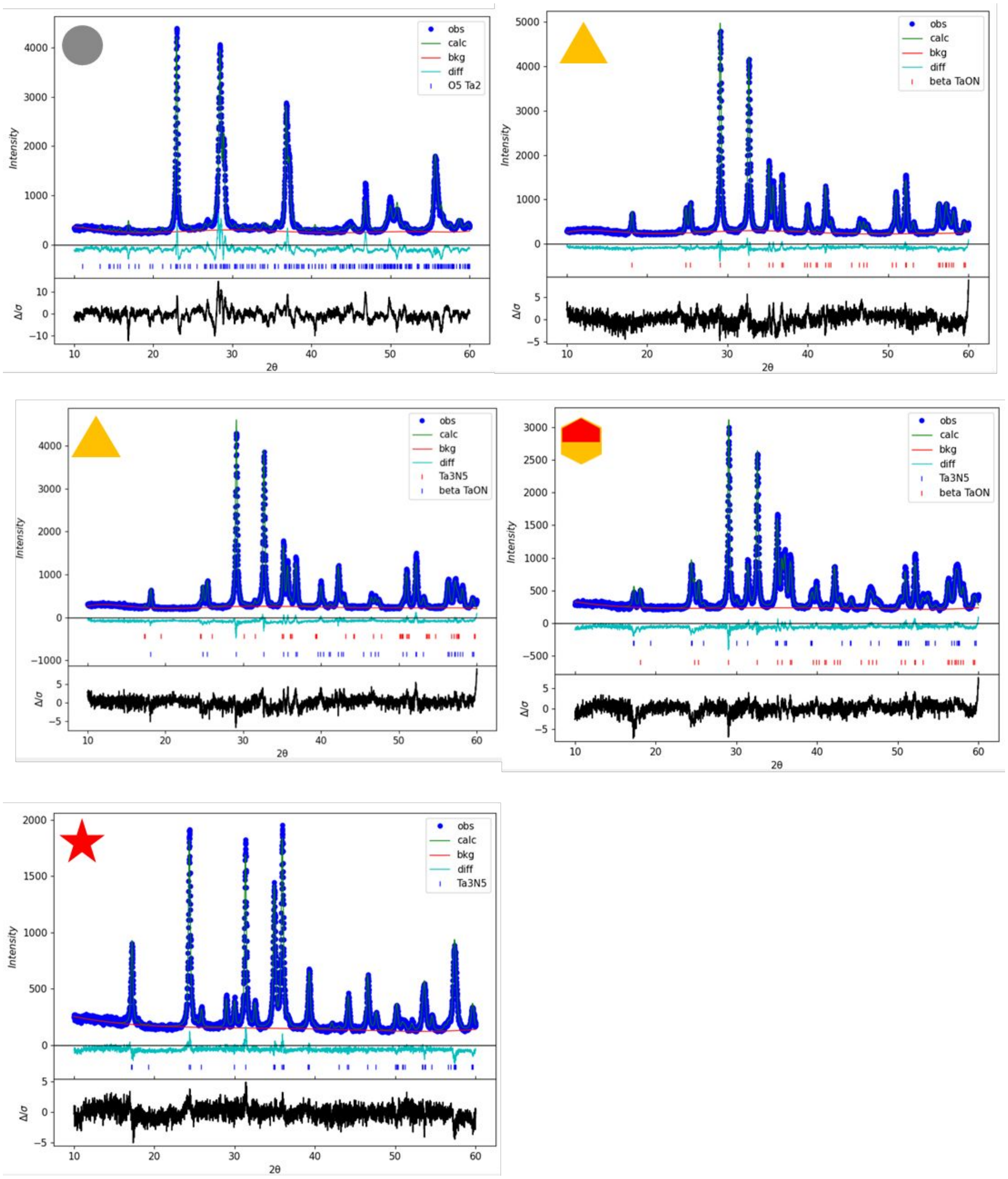

Figure S13. Measured (blue dots) and calculated (green line) XRD diffraction data, the latter from Rietveld refinement, for 1100 K reaction temperature products at higher water partial pressures (gas mixture containing NH_3, H_2O via Ar, and N_2). Symbols correspond to the respective products in the phase diagram (moving from lower to higher ammonia partial pressures). Refer to **Figure 5f** in main manuscript.

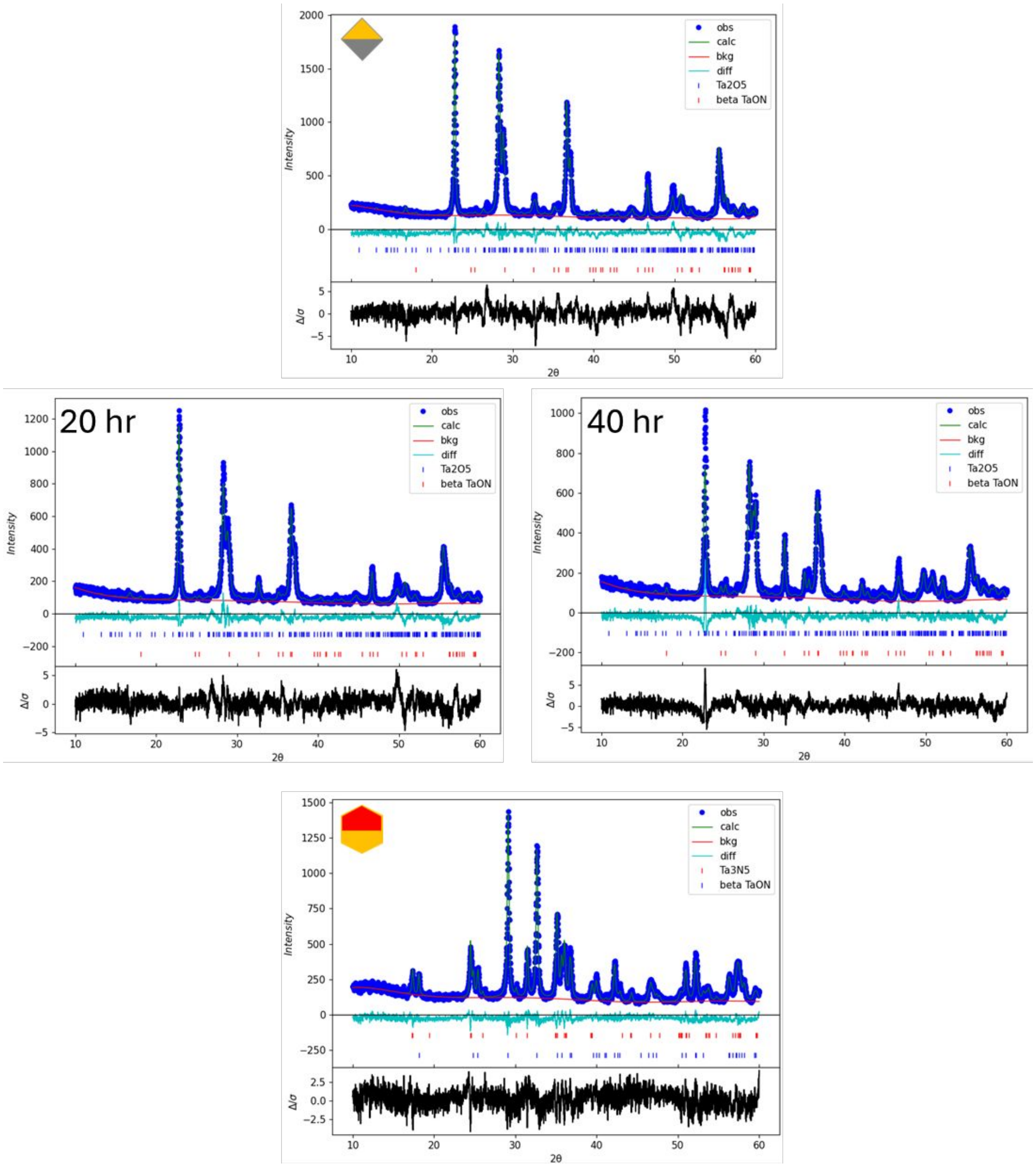

Figure S14. Measured (blue dots) and calculated (green line) XRD diffraction data, the latter from Rietveld refinement, for 1050 K reaction temperature products with gas mixture containing NH_3 and H_2O via Ar. Symbols correspond to the respective products in the phase diagram (moving from lower to higher ammonia partial pressures). Refer to **Figure 6b** in main manuscript.

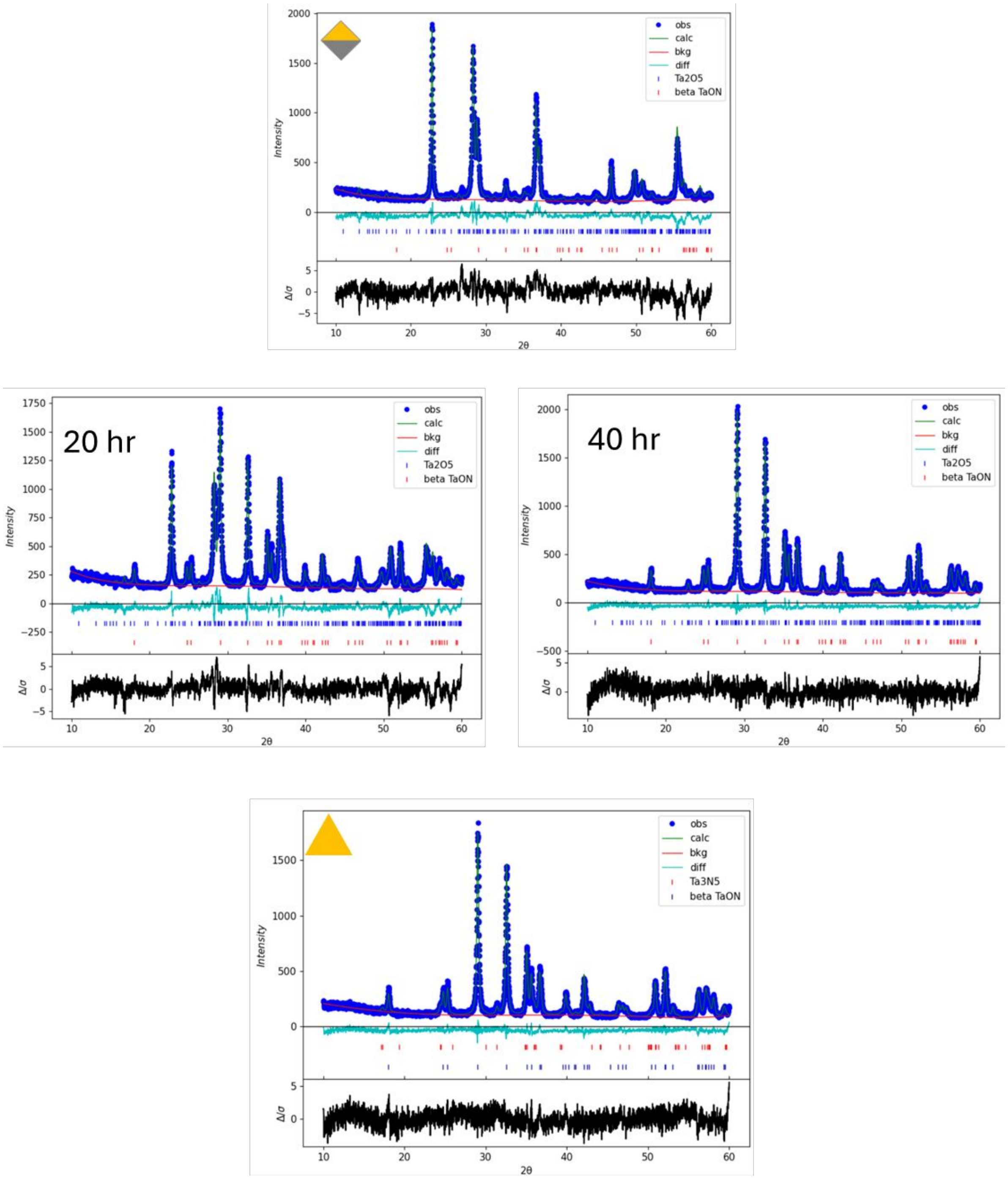

Figure S15. Measured (blue dots) and calculated (green line) XRD diffraction data, the latter from Rietveld refinement, for 1100 K reaction temperature products with gas mixture containing NH_3 and H_2O via Ar. Symbols correspond to the respective products in the phase diagram (moving from lower to higher ammonia partial pressures). Refer to **Figure 6d** in main manuscript.

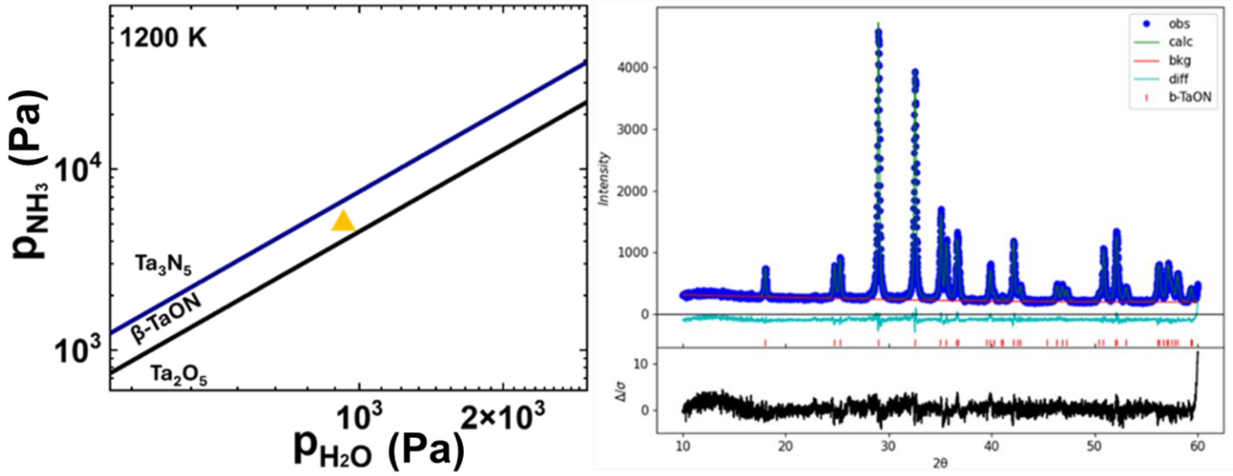

Figure S16. Computationally-predicted Ellingham-type diagram of β–TaON synthesis window at 1200 K with experimental synthetic validation (left). Measured (blue dots) and calculated (green line) XRD diffraction data, the latter from Rietveld refinement, for 1200 K reaction temperature product with gas mixture containing N_2, NH_3 and H_2O via Ar (right). Refinement yields product phase fraction of 100% β–TaON.

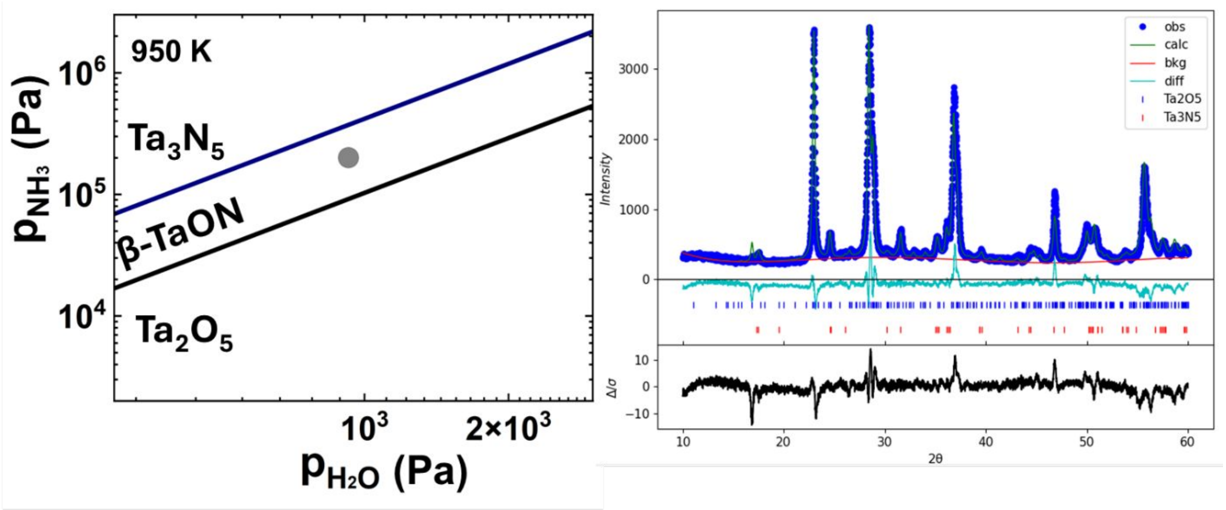

Figure S17. Computationally-predicted Ellingham-type diagram of β–TaON synthesis window at 950 K with experimental synthetic validation (left). Measured (blue dots) and calculated (green line) XRD diffraction data, the latter from Rietveld refinement, for 950 K reaction temperature product with gas mixture containing N_2, NH_3 and H_2O via Ar (right). Refinement yields a product phase fraction of 78% Ta_2O_5 + 22% Ta_3N_5.

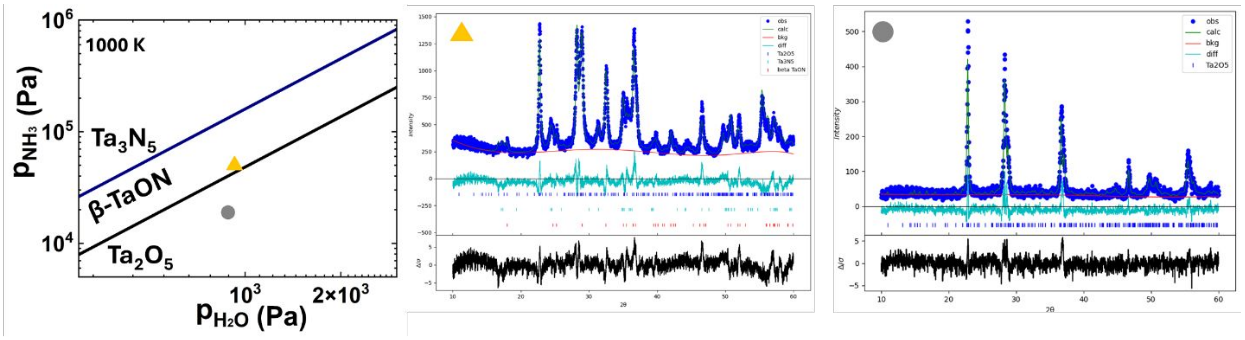

Figure S18. Computationally-predicted Ellingham-type diagram of β–TaON synthesis window at 1000 K with experimental synthetic validation (left). Measured (blue dots) and calculated (green line) XRD diffraction data, the latter from Rietveld refinement, for 1000 K reaction temperature products with gas mixture containing N_2, NH_3 and H_2O via Ar (middle and right). Refinement yields product phase fractions of 76% β-TaON + 14% Ta_2O_5 + 10% Ta_3N_5 (middle) and 100% Ta_2O_5 (right).

Table S1 Benchmark of DFT-computed ZPE and thermal corrections for N_2 and O_2

	**Property**	**This work**	**Reference**	**Difference**
N_2	ZPE(eV)	0.150	0.146[3]	3.0%
O_2	ZPE(eV)	0.097	0.098[3]	0.5%
O_2	ZPE+thermal correction at 298.15K (eV)	-0.4506	-0.4467[4]	0.9%

Table S2 Formation enthalpy of Ta compounds calculated using different methods, in comparison with values measured experimentally[5] and finalized through the computational–experimental feedback loop in the present work. The unit is kJ/(mol•atom).

	$\Delta H_f^{298.15K}$	$\Delta H_f^{0K,DFT}$	$\Delta H_f^{0K,DFT,correct}$	ΔH_f^{rxn}	ΔH_f^{expt}	ΔH_f^{final}
β-TaON	-186.33	-186.99	-223.22	-214.58	N/A	-193.41
Ta_3N_5	-103.07	-102.96	-129.86	-113.65	N/A	-100.09
Ta_2O_5	-246.22	-245.76	-292.63	N/A	-292.72	-292.72
TaN	-111.95	-111.86	-133.40	N/A	-126.15	N/A

In addition to their values derived using the reaction-based method, the formation enthalpies of solid phases were also calculated using the pure elements as the references. Specifically, the

formation enthalpies were calculated by adopting different reference state energies for the gas molecules, including (1) 298.15 K enthalpies ($\Delta H_f^{298.15\text{K}}$) obtained by including the zero-point energies (ZPE) and thermal corrections from translational, rotational, and vibrational contributions (**Table S1**); (2) 0 K enthalpies ($\Delta H_f^{0\text{K,DFT}}$)obtained from standard GGA-PBE calculations; (3) Empirically corrected GGA-PBE 0 K enthalpies ($\Delta H_f^{0\text{K,DFT,correct}}$) to account for the known limitations of GGA in accurately describing exchange-correlation effects in metal oxides and nitrides and gas-molecule binding energetics[6,7]. In addition to β-TaON and Ta_3N_5, we also extended these calculations to Ta_2O_5 and TaN, of which experimental formation enthalpies are available for validating the calculated values. The calculation results are summarized in **Table S2**.

As shown in **Table S2**, the differences between $\Delta H_f^{0\text{K,DFT}}$ and $\Delta H_f^{298.15\text{K}}$ of the oxide, nitride and oxynitride phases are very small relative to the enthalpy adjustments determined by the computational–experimental feedback loop. This indicates that ZPE and thermal corrections alone cannot account for the discrepancy between the DFT-predicted formation enthalpies and experimentally refined values.

Moreover, it is worth noting that both $\Delta H_f^{298.15\text{K}}$ and $\Delta H_f^{0\text{K,DFT}}$ for Ta_2O_5 and TaN deviate substantially from the experimental values (ΔH_f^{expt}). This is not surprising, as the GGA method is known to have limitations in accurately describing exchange-correlation effects in metal oxides and nitrides, as well as the binding energetics of gas molecules[6,7]. For example, the GGA method often overbinds gas molecules and thus predicts overly negative binding energies. To partially correct this error, Wang et al. proposed a correction of -1.36 eV per O_2 molecule for DFT-predicted formation enthalpies. This correction was determined by fitting DFT results to experimental formation enthalpies of a set of non-transition metal oxides and was intended primarily to account for the gas-molecule overbinding error. When applied to transition metal oxides, however, its accuracy becomes system dependent[6]. Using a similar fitting procedure, Grindy et al. reported a value of -0.892 eV per N_2 molecule for nitride formation enthalpy corrections[7].

The formation enthalpy of Ta compounds obtained using these empirical corrections are listed in the $\Delta H_f^{0\text{K,DFT,correct}}$ column of **Table S2**. Although the corrected value for Ta_2O_5 agrees well with the experimental result, the corrected value for TaN remains substantially inaccurate. In addition, the corrected formation enthalpies of both β-TaON and Ta_3N_5 still deviate significantly from the values predicted by the CALPHAD model finalized by the computational–experimental feedback loop. In other words, using $\Delta H_f^{0\text{K,DFT,correct}}$ as the initial input would require even larger enthalpy adjustments than using the reaction-based values, ΔH_f^{rxn}, adopted in the present work.

The discrepancies among the formation enthalpies obtained using different calculation methods further highlight that DFT alone cannot accurately predict the formation enthalpy of Ta oxides, oxynitrides, and nitrides and therefore the phase equilibria among them. Experimental input is

essential for finalizing a thermodynamic model capable of making reliable predictions. As a starting point, we used $\Delta H_f^{\mathrm{rxn}}$ as the initial value for the further refinement based on computation-experiment feedback loop.

Reference:

(1) Meisner, K. J.; Zaman, R.; Zhou, B.-C. Thermodynamic Modeling of the Ta-O System. *Calphad* **2022**, *76*, 102391. https://doi.org/10.1016/j.calphad.2022.102391.
(2) Jacob, K. T.; Shekhar, C.; Waseda, Y. An Update on the Thermodynamics of Ta2O5. *The Journal of Chemical Thermodynamics* **2009**, *41* (6), 748–753. https://doi.org/10.1016/j.jct.2008.12.006.
(3) Irikura, K. K. Experimental Vibrational Zero-Point Energies: Diatomic Molecules. *Journal of Physical and Chemical Reference Data* **2007**, *36* (2), 389–397. https://doi.org/10.1063/1.2436891.
(4) Wang, V.; Xu, N.; Liu, J. C.; Tang, G.; Geng, W.-T. VASPKIT: A User-Friendly Interface Facilitating High-Throughput Computing and Analysis Using VASP Code. *Computer Physics Communications* **2021**, *267*, 108033. https://doi.org/10.1016/j.cpc.2021.108033.
(5) Börnstein, L. *Thermodynamic Properties of Inorganic Material, Scientific Group Thermodata Europe (SGTE)*; Springer-Verlag: Berlin-Heidelberg, 1999.
(6) Wang, L.; Maxisch, T.; Ceder, G. Oxidation Energies of Transition Metal Oxides within the GGA + U Framework. *Phys. Rev. B* **2006**, *73* (19), 195107. https://doi.org/10.1103/PhysRevB.73.195107.
(7) Grindy, S.; Meredig, B.; Kirklin, S.; Saal, J. E.; Wolverton, C. Approaching Chemical Accuracy with Density Functional Calculations: Diatomic Energy Corrections. **2013**.
